# The Role of CD4^+^ T Helper Cell Subsets in Hepatocellular Carcinoma: Implications for Tumour Progression and Immunotherapy

**DOI:** 10.3390/cells15040350

**Published:** 2026-02-15

**Authors:** Jijie Shao, Jintong Na, Honghua Huang, Lei Xiao, Fengqiu Dang, Rongshun Zheng, Liping Zhong, Yongxiang Zhao

**Affiliations:** 1 The First Clinical Medical College, Guangxi Medical University, Nanning 530021, China; 2 State Key Laboratory of Targeting Oncology, National Center for International Research of Bio-Targeting Theranostics, Guangxi Key Laboratory of Bio-Targeting Theranostics, Collaborative Innovation Center for Targeting Tumor Diagnosis and Therapy, Guangxi Talent Highland of Major New Drugs Innovation and Development, Targeting Theranostics Research Center of Guangxi Higher Education, Guangxi Medical University, Nanning 530021, China; 3 Pharmaceutical College, Guangxi Medical University, Nanning 530021, China

**Keywords:** hepatocellular carcinoma, T helper cells, immunotherapy, regulatory T cells, Th/Treg balance

## Abstract

Hepatocellular carcinoma (HCC) remains one of the leading causes of cancer-related mortality; its progression is strongly linked to the liver’s immune microenvironment. T-helper (Th) cells, including Th1, Th2, Th17, and regulatory T cells (Tregs), play pivotal roles in modulating tumour immunity, either promoting or inhibiting tumour growth depending on their functional states and interactions within the tumour microenvironment. Imbalances in Th cell subsets, particularly between pro-inflammatory and immunosuppressive populations, have been associated with HCC progression and poor prognosis. Numerous studies have explored the therapeutic potential of restoring balance among Th cell subsets, focusing on modulating immune responses to improve HCC treatment outcomes. This paper reviews the differentiation and functional roles of Th cell subsets in HCC, exploring their contributions to tumour progression and immune suppression. Furthermore, this study discusses emerging immunotherapies aimed at modulating Th cell populations to improve clinical outcomes for HCC patients. Understanding the intricate roles of Th cells in the tumour microenvironment provides valuable insights for developing novel therapeutic strategies for liver cancer.

## 1. Introduction

Liver cancer is one of the most aggressive malignancies globally, ranking as the sixth most common cancer by incidence and the third by mortality. According to 2022 global cancer statistics, over 860,000 new cases of liver cancer are diagnosed annually, with 750,000 fatalities recorded. The disease warrants attention due to its low survival rates and high recurrence, with the majority of patients diagnosed at intermediate or advanced stages [1,2]. Primary liver cancer is primarily composed of three types: hepatocellular carcinoma, which accounts for 75–85% of cases, intrahepatic cholangiocarcinoma (ICCA), which represents 10–15%, and the rare combined hepatocellular–cholangiocarcinoma form [3]. The primary cause of liver cancer is chronic inflammation, often induced by hepatitis B (HBV) and C (HCV) viruses. Over 50% of HCC cases arise in a persistent inflammatory microenvironment [4], which drives tumour initiation and progression through mechanisms like the activation of proliferation and survival pathways, induction of angiogenesis, immune evasion, cancer stem cell maintenance, metastasis, and genomic instability. These processes significantly complicate treatment, compounded by the cancer’s evasion of the immune system [5,6,7]. For early-stage liver cancer, curative treatments such as local ablation, surgical resection, and liver transplantation can be effective. In advanced cases, immunotherapies, including kinase inhibitors and immune checkpoint inhibitors, offer more effective treatment options and can enhance survival rates for patients with recurrent tumours [8,9,10]. Research indicates that the immune cell populations in the livers of healthy individuals differ significantly from those of patients with hepatocellular carcinoma, suggesting that immune cells play a crucial role in the progression and treatment of liver cancer [11,12].

Th cells are central to both innate and adaptive immune responses and are vital components of the tumour microenvironment (TME). These cells have drawn increasing attention in tumour immunology and immunotherapy. Upon encountering various stimuli, Th cells adapt and mount appropriate immune responses, contributing significantly to the functioning of the body’s immune system [13,14]. Depending on the cytokine environment, naive CD4^+^ T cells differentiate into five major Th cell subsets: Th1, Th2, Th17, Tfh, and Tregs [14]. These subsets are strongly associated with tumour progression. Moreover, abnormal activation of Tregs is one of the primary drivers of tumour immune evasion [15,16,17]. Alterations in the number of CD4^+^ T cells and their subsets, particularly Th cells, as well as shifts in immune profiles, can influence the progression of hepatocellular carcinoma. Effective anti-tumour immunity requires the active involvement of Th cells [18,19]. This review provides an in-depth analysis of Th cell origin, differentiation pathways, and the specific roles of each subset in liver cancer. It also proposes therapeutic strategies targeting Th cells to improve liver cancer treatment outcomes.

## 2. Differentiation of CD4^+^ T Cells

### 2.1. Cellular Developmental Stages

CD4^+^ T cells have been demonstrated to play a crucial role in the adaptive immune system, representing a subset of T lymphocytes. T lymphocytes originate in the bone marrow, but their primary site of development is the thymus. Within the thymus, haematopoietic stem cells (HSCs) or common lymphoid progenitor cells (LPCs) migrating from the bone marrow or foetal liver undergo a series of double-negative (DN1-4) selection phases (DN1: CD44^+^CD25^−^; DN2: CD44^+^CD25^+^; DN3: CD44^−^CD25^+^; DN4, CD44^−^CD25^−^), followed by a double-positive selection phase (DP; CD4^+^CD8^+^) during which rearrangement occurs to generate CD4^+^CD8^+^ double-positive thymocytes (DP) [20,21]. At this stage, cells undergo dual selection through positive and negative selection. Positive selection ensures that T cells recognise self-major histocompatibility complex (MHC) molecules. Negative selection eliminates T cells with high affinity for self-antigens, thereby preventing autoimmune reactions. Subsequently, during DP cell development within the thymus, CD4^+^ and CD8^+^ T cells receiving optimal TCR signals undergo positive selection in the thymic cortex, yielding CD4^+^ or CD8^+^ single-positive (SP) T cells [20,22]. Through these selection processes, the retained CD4^+^ T cells acquire the capacity to recognise foreign antigens while avoiding attacks on self-tissues, ultimately emerging as naive CD4^+^ T cells within the peripheral immune system.

### 2.2. Cell Differentiation Stages

The differentiation of CD4^+^ T cells into T-helper cells typically proceeds through three stages (naive T cell stage, antigen-activated stage, and effector T cell stage). During the naive T cell stage, mature CD4^+^ single-positive T cells in the periphery migrate to secondary lymphoid organs, where they await activation and are known as naive T cells [23]. Initial T cells exist in a quiescent state; their surface molecules—the T cell receptor (TCR)—are mature but not yet activated by antigens. They await antigen presentation by antigen-presenting cells [24]. Upon recognition by the TCR of a naive T cell of the MHC class II molecule complex on an antigen peptide on the surface of an antigen-presenting cell (APC), the CD4^+^ T cell is activated [25]. Quiescent T cells enter the antigen-activated phase, a process requiring two signals: the first signal arises from the binding of the TCR to the antigen peptide–MHC complex. This confers specificity to the immune response, ensuring only antigen-specific lymphocytes are activated and, therefore, initiating signal transduction pathways [26]. The second signal involves the interaction of co-stimulatory molecules, where co-stimulation is essential for the normal survival, activation, and differentiation of T cells. Co-stimulation primarily arises from the interaction of a series of co-stimulatory molecule pairs between T cells and antigen-presenting cells (APCs). The most crucial pair comprises the CD28 molecule (on the T cell surface) and the B7 molecule (on the APC surface). Upon binding between CD28 and B7, a co-stimulatory signal is transmitted into the T cell. This co-stimulatory signal enhances the T cell’s activated state. It synergises with the primary signal to achieve full T cell activation. In the absence of co-stimulatory signals, T cells typically enter a non-responsive state [27,28].

**Figure 1:**
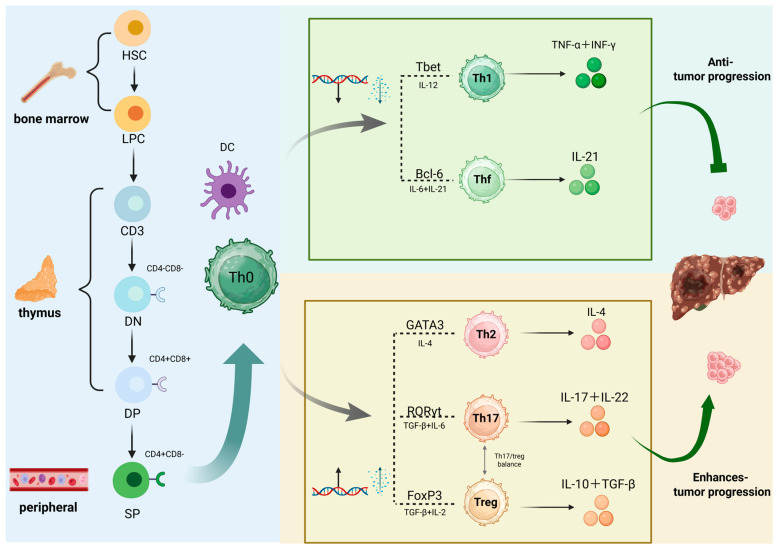
CD4^+^ T cell differentiation and its role in hepatocellular carcinoma progression. Haematopoietic stem cells in the bone marrow differentiate into lymphoid progenitor cells, which subsequently develop into mature T cells within the thymus. Primitive T cells differentiate into Th1, Th2, Th17, and Treg cells following stimulation by antigen-presenting cells [31]. Th1 cells suppress tumour growth by secreting TNF-α and IFN-γ, whereas Th2, Th17, and Treg cells promote tumour progression through secretion of IL-4, IL-17, IL-22, IL-10, and TGF-β.

Following antigen stimulation, naive CD4^+^ T cells undergo proliferation and, under the combined influence of the cytokine microenvironment and lineage-specific transcription factors, differentiate into multiple functional subsets through epigenetic reprogramming [29,30]. T-helper cells are typically classified into five major subsets (Figure 1): Th1 cells, which secrete interferon-γ (IFN-γ) and utilise T-bet as a key transcription factor; Th2 cells, which secrete interleukin-4, IL-5, and IL-13 and employ GATA3 as their primary transcription factor; Th17 cells secreting IL-17 and IL-22 with RORγt as the key transcription factor; T follicular helper cells (Tfh) secreting IL-21 with Bcl6 as the primary transcription factor; and Tregs secreting IL-10, TGF-β and IL-35 with FOXP3 as the key transcription factor [31]. Furthermore, some studies have described other subsets, such as Th9 cells, which secrete IL-9 [32] and Th22 cells, which secrete IL-22 [33]. Following a series of regulatory processes and developmental stages, the activated and differentiated T helper cells migrate to their effector sites to perform their functions.

### 2.3. Immunoregulation of CD4^+^ T Cells by the Liver Immune Microenvironment

The liver, as a classic immunologically privileged organ, possesses unique immunoregulatory properties and a specialised blood supply system. Its primary blood source is the terminal portal vein, where circulating lymphocytes in the blood come into contact with resident hepatocytes at the hepatic sinusoids. Liver sinusoidal endothelial cells (LSECs), functioning as porous endothelium, separate blood from hepatocytes to form the Disse space barrier, providing attachment opportunities for leukocytes. Under low perfusion pressure, leukocytes can adhere without requiring selectin molecules. Resident APCs interact with circulating lymphocytes, thereby creating unique conditions for T cell regulation [34,35].

The hepatic microenvironment plays a crucial role in the differentiation and functional regulation of CD4^+^ T cells. In inflammatory conditions such as hepatitis, hepatocytes acquire the ability to present MHC class II molecules, thereby activating CD4^+^ T cells. Concurrently, HSCs promote Treg differentiation by secreting retinoic acid and TGF-β and enhancing immune tolerance and suppressing immune responses [36,37]. Interestingly, naive CD4^+^ T cells in the liver typically differentiate into Th2 subsets rather than Th1 or Th17 subsets, a phenomenon termed clonal drift. Furthermore, LSECs exert an inhibitory effect on the activation of naive CD4^+^ T cells, failing to promote Th1 cell differentiation even in the presence of exogenous IL-1β, IL-12, and IL-18 [38,39]. Kupffer cells, acting as APCs in the liver, can produce IL-12 and IL-27 through the CD40-CD40L signalling pathway These cytokines promote the expansion of the CD4^+^ T cell pool and the activation of T cells [40,41]. These mechanisms collectively shape the liver’s unique immune microenvironment and influence the intensity and type of immune responses.

## 3. The Role of Tregs in Hepatocellular Carcinoma: Tumour Promotion and Therapeutic Implications

### 3.1. The Promoting Role of Tregs in Hepatocellular Carcinoma

**Figure 2:**
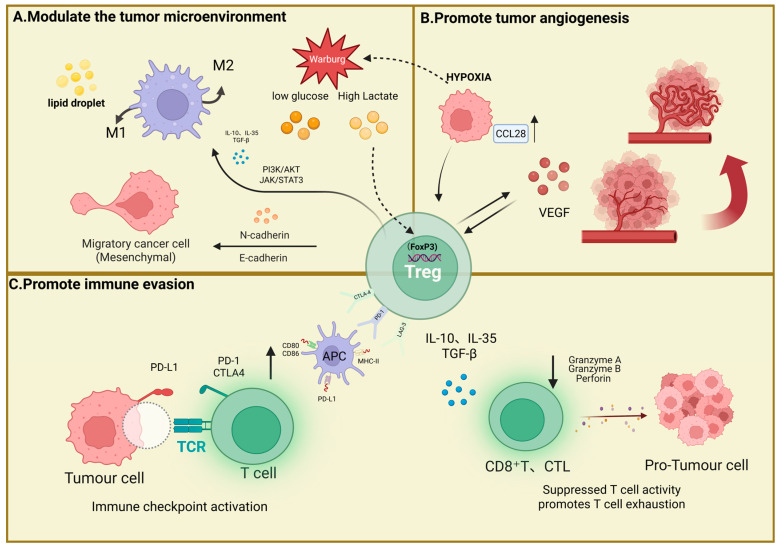
Mechanisms of Treg cells in the tumour microenvironment. (**A**) Tregs modulate the tumour microenvironment via IL-10, IL-35, and TGF-β, influencing M1/M2 macrophage polarisation and tumour cell migration [44]. (**B**) Tregs promote tumour angiogenesis by secreting CCL28 and VEGF [63,64]. (**C**) Tregs induce immune evasion by suppressing T cell activity via PD-1/CTLA4 inhibition, thereby facilitating tumour cell proliferation. The synergistic actions of these signalling pathways and cytokines collectively influence tumour progression [50,51].

Treg cells play a pivotal regulatory role within the immunosuppressive microenvironment of hepatocellular carcinoma. Tregs are a specialised subset of T helper cells characterised by high expression of the IL-2 receptor α chain CD25. Forkhead Box P3 (FOXP3), a regulator belonging to the Forkhead/winged worm family, is a key factor in the regulation of Treg growth and function [42]. Studies have shown that Treg cells drive tumour progression in hepatocellular carcinoma and suppress immune responses through multiple mechanisms. Their functional network comprises complex processes such as immune escape, angiogenesis regulation, and metabolic competition (Figure 2). Treg cell counts have also been demonstrated to serve as a prognostic biomarker and indicator of recurrence for hepatocellular carcinoma [43].

In terms of immune evasion, Tregs directly attenuate the proliferation, cytokine production, and cytotoxic activity of effector T cells through direct contact or secretion of inhibitory cytokines (IL-10, TGF-β, and IL-35), thereby establishing a local immunosuppressive microenvironment [44]. Multiple studies have shown that in patients with hepatocellular carcinoma, the number of regulatory T cells in tumour-infiltrating lymphocytes and peripheral blood is increased. Studies have shown that Tregs not only inhibit apoptosis and degranulation of CD8^+^ T cells, granzymes A and B, and apoptin, but also similarly inhibit apoptosis, activation, degranulation, and cytokine production in CD4^+^ cells [45,46,47]. As a result, pore formation on target cell membranes is significantly reduced, leading to diminished killing efficiency of CTLs and NK cells. Consequently, tumour cells evade immune surveillance, thereby achieving immune escape [48]. Yang, M. et al. employed spatial proteomics to compare the immune microenvironments of primary and recurrent hepatocellular carcinoma, revealing that increased regulatory T cells and T cell exhaustion mutually reinforce each other and simultaneously exacerbate immunosuppression, promoting immune evasion [49]. This study further revealed that in liver cancer, peripherally derived Tregs upregulate immune checkpoints such as CTLA-4, resulting in preferential accumulation of CTLA-4 in tumour-infiltrating Tregs and exhausted CD8^+^ T cells. Studies have shown that Tregs can further induce PD-L1 expression through secretory factors, ultimately reducing the efficacy of PD-1/PD-L1 inhibitors [50,51]. Concurrently, miR-500a-3p carried by HCC-derived exosomes promotes the differentiation of CD4^+^ T cells into Tregs by upregulating PD-1 expression, thereby enhancing the immunosuppressive microenvironment [52].

In terms of cellular metabolic regulation, dysregulation of metabolic pathways helps cancer cells adapt to unfavourable microenvironments, thereby supporting their rapid expansion [53]. Liver cancer cells undergo metabolic reprogramming, exhibiting the Warburg effect, which elevates their glycolytic activity. Consequently, they perform glycolysis even under aerobic conditions, producing substantial amounts of lactic acid [54,55]. Lactic acid increases FOXP3 expression and enhances Treg cell functions, enabling them to maintain stability and functionality within inflammatory environments [56]. Concurrently, the FOXP3 gene can reprogramme T cell metabolism, enabling them to function effectively in environments characterised by low glucose and high lactate levels [57]. Elevated lactate and reduced glucose conditions in liver cancer cells prevent CD8^+^ T cell infiltration while promoting the accumulation of FOXP3^+^ Tregs. This process generates lactate via the activin/SMAD/LDHA axis, thereby facilitating the recruitment and accumulation of Tregs within tumour tissue [58]. Tregs may also promote hepatocellular carcinoma progression via lipid metabolism. Wang et al. demonstrated that hepatocellular carcinoma induces lipid metabolic reprogramming in monocytes/macrophages, leading to lipid-droplet accumulation. This process mediates macrophage survival and Treg recruitment through the CCL20/CCR6 axis [59]. Moreover, Zhou, X et al. discovered through exploratory research into gene prognostic models that neoplastic adipogenesis promotes Treg recruitment in hepatocellular carcinoma and fosters an immunosuppressive microenvironment [60]. Tregs can also influence macrophage polarisation and metabolism through specific signalling pathways, thereby reducing the number of M1 macrophages in hepatocellular carcinoma and inducing the polarisation of M2 macrophages [61]. Tregs can also enhance the stemness of HCC cells by upregulating tumour-initiating cell (TIC)-associated markers, such as CD133, inducing epithelial-to-mesenchymal transition (EMT), increasing the TIC ratio, and promoting tumorigenicity [62].

In the process of regulating angiogenesis, studies have shown that Tregs themselves promote the production of vascular endothelial growth factor (VEGF). Under hypoxic conditions, liver tumours recruit Tregs by upregulating CCL28 expression. The increased VEGF levels in Treg cells under hypoxic conditions promote microvascular angiogenesis within tumour tissue, thereby enhancing the supply of nutrients and oxygen for tumour growth and proliferation [63,64]. Concurrently, elevated VEGF levels stimulate the proliferation and accumulation of Treg cells. Tumour-derived VEGF acts as a chemotactic factor, attracting Tregs to the tumour microenvironment where they exert their effects. Through signalling pathways such as STAT3, the immunosuppressive function of Tregs is enhanced, thereby promoting their differentiation and stability [65,66]. This mechanism fosters a mutually reinforcing relationship between VEGF and Tregs, thereby driving tumour progression.

### 3.2. Therapeutic Role and Prognostic Significance of Tregs in Hepatocellular Carcinoma

Research indicates that Tregs serve as an independent and quantifiable adverse prognostic indicator for HCC. Multicentre retrospective cohort studies and single-cell sequencing reveal that an increase in intratumoural Treg cells correlates with heightened disease risk in patients, and the FOXP3^+^ Treg subset is recognised as an independently detectable risk factor [67,68]. Moreover, the quantity and spatial distribution of Treg cells, as assessed through multiplex quantitative immunofluorescence and single-cell RNA sequencing analysis, have been shown to exert differential effects on the long-term prognosis of HCC. and are better predictors of recurrence in early-stage liver cancer [69]. Mechanistically, HCC cells secrete TGF-β to induce peripheral-naive CD4^+^ T cells and polarise towards Tregs. Elevated TGF-β levels exhibit a linear negative correlation with the proportion of peripheral FOXP3^+^ Tregs, collectively reducing overall survival [68,70,71]. Furthermore, this study found that Tregs affect the balance of the immune system through the FOXO1-Th17/Treg mechanism, mediating immune dysfunction and promoting the development of HCC [72].

At the therapeutic level, multiple established conventional treatments have demonstrated clinical efficacy, including transarterial chemoembolisation (TACE), stereotactic body radiotherapy (SBRT), immunotherapy (IO), and thermal ablation techniques such as radiofrequency ablation (RFA). TACE and SBRT primarily induce DNA damage through ischaemia or radiation; RFA activates APCs by promoting the release of heat-shock proteins (HSPs) from thermal stress and exposing heat-modified tumour antigens. This triggers a systemic anti-tumour T cell response, creating an “in situ vaccination” effect [73,74,75,76]. Research indicates that radiofrequency ablation (RFA) significantly upregulates the expression of Th1-type cytokines (such as IL-2 and IFN-γ) within the tumour microenvironment, whilst simultaneously suppressing the production of the immunosuppressive factor IL-10, thereby reshaping the immune balance [77]. Further research indicates that remodelling the function of Tregs can promote the restoration of immune homeostasis in peripheral blood and the tumour microenvironment following treatment [78]. As a result, compared to simple static counting, dynamically monitoring and intervening in the function and distribution of Tregs may be a key strategy for further enhancing treatment outcomes and improving long-term prognosis in HCC patients.

## 4. The Role of T-Helper Cells in Hepatocellular Carcinoma Development and the Underlying Mechanisms

### 4.1. The Role of Th1 Cells in Hepatocellular Carcinoma

During the HCC process, Th1 cells release cytokines such as IFN-γ, IL-2, and TNF-α [79]. These cytokines transform the tumour microenvironment from cold to hot, rapidly activating the immune system to mount a systemic anti-tumour response. Research indicates that Th1 cells establish a favourable immune microenvironment by promoting the polarisation of macrophages toward the M1 phenotype. This enhances the activity of natural killer (NK) cells, enabling them to recognise and eliminate cancer cells with greater precision and efficacy. Concurrently, it stimulates the proliferation and differentiation of cytotoxic T lymphocytes (CTLs), elevating their specific cytotoxic capacity against cancer cells [80,81].

IFN-γ as the most emblematic effector molecule of Th1 cells, combats tumours through multiple synergistic mechanisms. It induces cell cycle arrest and apoptosis in cancer cells while inhibiting HBV replication, thereby severing the chain of “virus–inflammation–carcinogenesis” [82,83]. Inhibiting angiogenesis in newly emerging liver cancer while promoting maturation of residual blood vessels, and weakening tumour blood supply, improves the tumour microenvironment and suppresses tumour dissemination [84]. Research indicates that IFN-γ may synergistically interact with sorafenib to induce ferroptosis or collaborate with IL-12 to enhance the infiltration of tumour-infiltrating IFN-γ^+^CD8^+^ T cells, thereby further amplifying immune-mediated killing effects [85]. Interestingly, activated Th1 cells and NK cells can jointly secrete IFN-γ by promoting CXCL10 expression, thereby upregulating the receptor CXCR3. Upon activation, the CXCR3 receptor enhances Th1 polarisation while inhibiting the differentiation of Th0 cells into Tregs. This drives Th0 cells towards polarisation with Th1, establishing an IFN-γ-induced CXCL10/CXCR3 pathway that forms a positive feedback regulatory mechanism amplifying anti-tumour immunity [86].

Clinical data and animal model studies indicate that increased Th1 cell infiltration density in hepatocellular carcinoma tumour tissues correlates with longer overall survival in both patients and experimental animals [79,87]. Early screening trials using AFP-derived peptides reveal that anti-AFP Th1 responses are primarily observed in patients with early-stage hepatocellular carcinoma classified as Child–Pugh A. This suggests that Th1 responses may serve as biomarkers for early diagnosis and lymph node assessment [88]. These studies confirm that Th1 cells are closely implicated throughout the entire progression of hepatocellular carcinoma, enabling early warning of disease and indicating prognosis, while simultaneously inhibiting tumour progression and stabilising the tumour immune microenvironment.

### 4.2. The Role of Th2 Cells in Hepatocellular Carcinoma

Cytokines such as IL-4 cause naive CD4^+^ T cells to differentiate towards the Th2 pathway. Activated Th2 cells, through the autocrine secretion of IL-4, IL-5, IL-10, and IL-13, not only accelerate their own proliferation, but also suppress that of Th1 cells. Research indicates that Th2 cells establish a positive feedback loop that enhances Th2 responses by counteracting the differentiation and stability of Th1 cells and attenuating their IFN-γ-stimulated function [89,90]. While the tumour microenvironment of hepatocellular carcinoma exhibits Th2-polarised dominance, studies have found that this leads to reduced cytotoxic activity, thereby significantly diminishing the ability to kill target cells. Antigen presentation activity is lowered, preventing T cells from effectively recognising these antigens. Consequently, the initiation of the immune response fails, resulting in the establishment of an immunosuppressive microenvironment [91]. In the context of hepatocellular carcinoma arising from viral hepatitis, similar phenomena have been corroborated: an elevated proportion of peripheral Th2 cells in HCV-associated cirrhosis patients correlates with a synchronously increasing risk of subsequent hepatocellular carcinoma development [92]. HBV-driven HCC is linked to the expansion of peripheral Th2 subsets, as shown by studies using whole-exome sequencing and proteomic analysis, which revealed changes in immune cell populations and signalling pathways within the tumour microenvironment [93].

At the mechanistic level, Th2 responses may enhance pro-tumour effects through the following pathways: Th2-derived IL-4 directly drives tumour-associated macrophages towards polarisation with the M2 phenotype, subsequently releasing pro-angiogenic factors and matrix remodelling enzymes [94]. Persistent Th2 cytokine signalling increases tumour vascular infiltration and the probability of extrahepatic metastasis [95]. Recent studies further revealed that when tumour cells undergo necrosis, the substantial release of potassium ions leads to local accumulation. High concentrations of K^+^ rewire the metabolic profile of T cells, deliberately favouring the differentiation of Th2 and Treg cells while simultaneously suppressing the formation of the anti-tumour Th1 subset [96].

Clinical studies indicate that an elevated number of Th2 cells in peripheral blood is typically associated with later tumour stages and poorer treatment response. Prior to TACE therapy, patients with higher Th2 ratios are more prone to early progression; conversely, the absence of Th2 gene signatures in tumour tissue is significantly correlated with long-term patient survival [74,87]. Compared to healthy liver samples, most immune cell subsets required for anti-tumour immune responses were reduced in hepatocellular carcinoma samples, and the gene signatures of T helper cells and Th2 cells were significantly increased [87,97].

### 4.3. The Role of Th9 Cells in Hepatocellular Carcinoma

Th9 cells, as a newly identified subset of Th cells, exhibit complex roles in hepatocellular carcinoma. Initially identified as T cell growth factors with potential oncogenic activity, subsequent research has demonstrated that Th9 cells eliminate tumours by activating both innate and adaptive immune cells [98,99].

Th9 cells exert their effects by secreting the cytokine IL-9, which has potential pro-inflammatory effects [100]. IL-9 has been demonstrated to induce Th17 cell differentiation and enhance the function of FOXP3^+^ natural regulatory T cells [101]. Studies in HCC patients have revealed that IL-9 promotes hepatocellular carcinoma cell proliferation and metastasis, as well as VEGF expression, by activating the JAK2/STAT3 pathway [102]. Tan et al. found that co-culturing primary HCC cells with autologous Th9 cells significantly increased CCL20 production in tumour cells. Th9 cells secrete CCL20, thereby inducing epithelial–mesenchymal transition-like changes in HCC cells and promoting the progression of hepatocellular carcinoma [103,104]. Moreover, compared to peritumoural hepatic tissue, the expression levels of IL-9R and IL-9 were significantly elevated within tumour tissue. High IL-9R expression promotes the progression of hepatocellular carcinoma and indicates poor clinical prognosis [105]. However, research also indicates that liver cancer can be treated by enhancing Th9 cells. Following drug-induced elevation of IL-9 levels, activated CD8^+^ T cells release IL-2 and granzyme B to suppress the growth of liver cancer within the body [106]. A single high-dose irradiation HCC whole-cell lysate vaccine suppresses the growth of mouse hepatocellular carcinoma via Th9 cell numbers [107].

### 4.4. The Role of Th17 Cells in Hepatocellular Carcinoma

**Figure 3:**
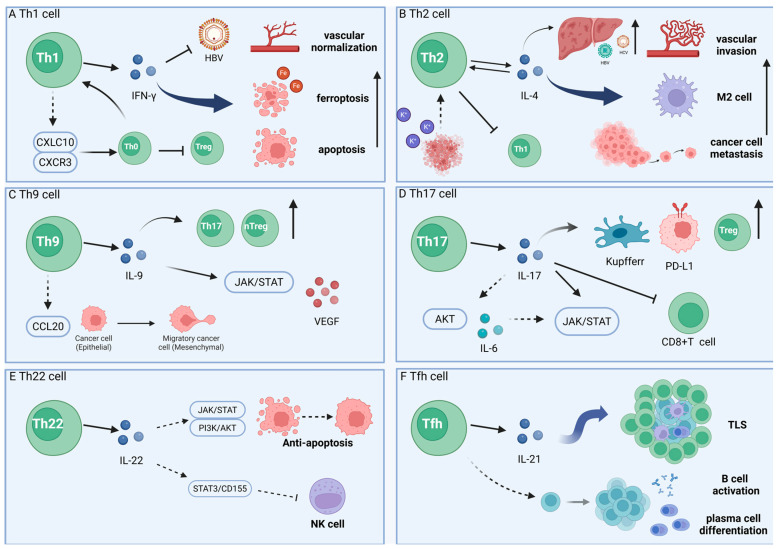
The role of different types of Th cells in the progression of hepatocellular carcinoma. (**A**) Th1 cells promote tumour cell apoptosis and vascular normalisation via IFN-γ [84]. (**B**) Th2 cells promote vascular invasion and tumour metastasis. (**C**) Th9 cells promote tumour cell growth and angiogenesis via VEGF [102]. (**D**) Th17 cells promote tumour cell growth by suppressing CD8^+^ T cell activity via PD-L1 [121]. (**E**) Th22 cells suppress NK cell function via the JAK/STAT signalling pathway [131]. (**F**) Tfh cells promote B cell activation and plasma cell differentiation via IL-21 [135,136].

Th17 cells are a subset of Th cells expressing the transcription factor RORγt. They primarily produce cytokines such as IL-17A, IL-17F, IL-22, and IL-21. These cytokines drive neutrophil recruitment and amplify inflammatory responses [108]. Under physiological conditions, Th17 cells reside in large numbers within the lamina propria of the small intestine. They reinforce the mucosal barrier while bridging innate and adaptive immunity to maintain microecological homeostasis [109]. However, in HCC, the sustained release of IL-17 can induce chronic inflammation. Numerous studies have highlighted the significant role of Th17 cells in HCC progression, linking them to enhanced angiogenesis, immunosuppression, and extracellular matrix remodelling, which are associated with poorer prognosis in HCC patients (Figure 3).

Numerous studies have revealed the pivotal role of Th17 cells in the carcinogenesis of hepatocellular carcinoma. One of the high-risk factors for inducing hepatocellular carcinoma is a shift in the balance of circulating Th cells from Th1 dominance to Th17 dominance during chronic HBV infection [110]. Previous studies have shown that the frequency of Th17 cells producing IL-17 in patients with HBV-associated HCC, as assessed through scRNA-seq and viral tracking to identify HBV-infected cells, is significantly higher compared to both patients with non-HBV-associated HCC and healthy controls [111]. Research indicates that non-alcoholic steatohepatitis, which is the liver’s unconventional pre-folded RPB5-interacting protein (URI), exhibits a unique mechanism of action for Th17 cells. It links nutritional surplus to inflammation and non-alcoholic steatohepatitis by leveraging Th17 cells and IL-17A to trigger inflammatory responses. This induces neutrophil infiltration in white adipose tissue, subsequently mediating insulin resistance (IR) and the release of fatty acids. These fatty acids are stored in the liver as triglycerides, ultimately leading to metabolic-associated steatohepatitis (MASH) [112]. In models of alcoholic fatty liver disease, IL-17 exacerbates hepatic lipid accumulation and promotes the secretion of multiple pro-inflammatory cytokines, thereby establishing a vicious cycle of steatosis–inflammation–carcinogenesis [113]. Th17 cells may also induce hepatocellular carcinoma through alternative mechanisms. Gasmi et al. showed that chronic exposure to IL-17 causes miR-122 downregulation in hepatic progenitor cells, reprogramming them into cancer stem cells, and thereby increasing the likelihood of liver cancer development [114].

As a crucial subset of Th cells, Th17 cells activate multiple signalling pathways through cytokine secretion, thereby driving the progression of hepatocellular carcinoma. Th17 cells activate HSCs by secreting IL-17 and promoting the development of liver fibrosis. Concurrently, they induce hepatocytes to secrete IL-6, which activates the STAT3 signalling pathway. This subsequently promotes tumour cell proliferation and inhibits apoptosis, thereby establishing a chronic inflammation-driven carcinogenic microenvironment [115]. Furthermore, IL-17 activates the AKT signalling pathway, which promotes the invasion–metastasis cascade, induces epithelial–mesenchymal transition (EMT), and facilitates HCC cell colonisation. Concurrently, it induces IL-6 production, which, in turn, activates the JAK2/STAT3 signalling pathway, synergistically accelerating the progression of hepatocellular carcinoma [116,117]. Research indicates that IL-22 can sustainably activate Th17 cells and increase STAT3 expression [118]. As a result, the upregulation of downstream targets leads to elevated levels of IL-8, MMP2, and VEGF, significantly promoting angiogenesis, neutrophil recruitment, and tumour growth in hepatocellular carcinoma [116]. Concurrently, within the hypoxic microenvironment of HCC, increased release of exosomes containing elevated levels of miR-4508 activates the IL17A-p38MAPK-κB signalling pathway in fibroblasts. This promotes the formation of pre-metastatic niches (PMNs), thereby enhancing the metastatic potential of hepatocellular carcinoma [119].

In terms of immune regulation, research has revealed that tumour-activated monocytes secrete key pro-inflammatory cytokines, triggering the proliferation of functional Th17 cells. Within HCC tissue, the accumulation of pro-inflammatory Th17 cells enables activated monocytes in the peritumoural stroma of hepatocellular carcinoma to promote the expansion of helper Th17 memory cells [120]. Th17 cell expansion further promotes Kupffer cell/macrophage activation and cholesterol synthesis in hepatocytes, significantly accelerating the incidence of hepatocellular carcinoma [113]. Activated monocytes/macrophages secrete inflammatory cytokines that induce tumour cells to express PD-L1. IL-17-activated monocytes promote PD-L1 upregulation through autocrine cytokines. These IL-17-stimulated monocytes exhibit significant inhibitory effects on cytotoxic T cell immunity in vitro [121]. IL-17A secreted by h17 cells further promotes PD-L1 expression on the surface of HCC cells, leading to resistance to anti-PD-L1 therapy. Combining anti-IL-17A with PD-L1 blockade significantly increases the infiltration of cytotoxic CD8^+^ T cells, which express high levels of interferon-γ, thereby reducing treatment resistance in HCC [122]. Concurrently, Th17 cells suppress CD8^+^ T cell responses by mediating TGF-β and inhibiting the proliferation of autologous CD8^+^ T cells [123]. This study further revealed that tumour cells promote elevated IL-17 production and Th17 cell proliferation in primary liver cancer through cell-to-cell contact. The increase in Th17 cells synchronously co-increases with Tregs and Bregs in primary hepatocellular carcinoma (PHC), thereby fostering an environment conducive to immune evasion [124].

Th17 cells also exert a significant influence on the treatment and recurrence of hepatocellular carcinoma. Gasmi et al. observed in a mouse animal model that IL-17 deficiency and anti-IL-17 therapy protected mice from liver tumour growth [114]. The overall survival (OS) and disease-free survival (DFS) of patients with higher densities of IL-17-producing cells were significantly shorter compared to those with lower densities. Specifically, patients with high peritumoural IL-17RE expression had a hazard ratio (HR) of 1.569 (95% CI: 1.315–1.873) for OS and a HR of 1.433 (95% CI: 1.234–1.663) for TTR, indicating a substantial increase in the risk of poor outcomes and recurrence [125]. In patients with primary hepatocellular carcinoma (PHC), the percentage of Th17 cells is positively correlated with tumour size, the portal vein tumour tract (PVTT), and particularly TNM staging [124]. Following the adoptive transfer of Th17 cells, the liver exhibited numerous large tumour nodules, indicating that Th17 cells possess properties that promote HCC recurrence. Th17 cells may exacerbate HCC recurrence by activating the epithelial–mesenchymal transition (EMT) programme, inducing cancer stemness and the formation of a pre-metastatic microenvironment alongside angiogenesis. Studies have shown that FOXO1 negatively regulates Th17 cells, which may play a role in reducing the recurrence of hepatocellular carcinoma [126].

### 4.5. The Role of Th22 Cells in Hepatocellular Carcinoma

Th22 cells, as a CD4^+^ T cell subset secreting IL-22 without releasing IL-17, exhibit expression regulated by the transcription factor aryl hydrocarbon receptor. Research has confirmed that IL-22, acting as a pro-inflammatory factor, serves as a crucial link between immune cells and tissue-resident cells during inflammatory episodes [127,128]. Th22 cells are known to play pivotal regulatory roles in the initiation, progression, and metastasis of hepatocellular carcinoma. Within hepatocellular carcinoma, IL-22 is primarily secreted by Th22 cells. IL-22 signalling within hepatocytes promotes HCC progression, enhances angiogenesis at established metastatic sites, influences cancer cell extravasation, and amplifies liver metastasis [129].

From the signalling pathway perspective, IL-22 may promote sorafenib resistance in HCC by activating the STAT3/CD155 signalling axis, thereby reducing tumour cell sensitivity to both sorafenib-mediated direct cytotoxicity and NK cell-mediated lysis [130]. Excessive IL-22 in the HCC microenvironment is known to promote tumour growth, inhibit apoptosis, and facilitate metastasis through STAT3 activation [118]. Furthermore, IL-22 can upregulate anti-apoptotic and metastatic genes in HCC via the JAK/STAT and PI3K/AKT signalling pathways [131]. This promotes hepatocyte proliferation within an environment of injury and inflammation, thereby providing the cellular basis for the development of hepatocellular carcinoma. Clinical studies have shown that HCC patients exhibit higher levels of peripheral blood Th22 cells and IL-22 compared to healthy individuals; these levels increase as the disease progresses [132]. High tumour-infiltrating IL-22 cell counts and serum IL-22 levels are recognised as poor prognostic indicators for HCC. Multivariate analysis has shown that tumour-infiltrating IL-22 cells and serum IL-22 levels are independent prognostic factors for OS and DFS, with hazard ratios (HRs) of 1.65 (95% CI: 1.34–2.05) for OS and 1.42 (95% CI: 1.20–1.68) for DFS, both significantly associated with reduced survival rates [133]. Increased Th22 cell numbers have been shown to promote the growth of invasive cancers and angiogenesis; the proportion of Th22 cells correlates with tumour lymph node metastasis (TNM) staging and intrahepatic metastasis in HCC patients [134].

### 4.6. The Role of Tfh Cells in Hepatocellular Carcinoma

Tfh Cells are specialised cells that assist B cells, playing a crucial role in the formation of germinal centres, antibody affinity maturation, and the development of high-affinity antibodies and memory B cells. They both promote B cell differentiation and antibody production to enhance humoral immune responses, and secrete IL-21 to drive B cell activation and differentiation into plasma cells (Figure 3) [135,136].

Elevated PD-1 and PD-L1 signalling in HCC induces Tfh-specific depletion. Tfh cell exhaustion leads to reduced IL-21 production, subsequently impairing B cell proliferation and differentiation of functions. This results in B cell immunodeficiency [137,138]. Kurebayashi et al. [139]. identified that, based on the spatial dynamics of T cell responses, the differentiation of follicular Tfh Cells correlates with the development of tertiary lymphoid structures (TLSs) in hepatocellular carcinoma. The presence of tumour-intrinsic tertiary lymphoid structures (iTLS–ectopic lymphoid aggregates formed under chronic inflammatory conditions) is associated with favourable clinical outcomes in patients with hepatocellular carcinoma [135,139,140]. Multiplex immunohistochemistry studies have revealed that tumour-associated lymphoid structures (TLSs) in hepatocellular carcinoma are rich in Tfh cells, which play a crucial role in the maturation of TLSs, supporting the formation and maintenance of germinal centres and activating effective anti-tumour immune responses [141]. Compared to healthy individuals and cancer-adjacent tissue, both peripheral blood and tumour-infiltrating Tfh cells are reduced in hepatocellular carcinoma patients. Furthermore, lower peripheral Tfh cell counts have been associated with poorer prognosis in these patients [95]. Tfh cells not only predict prognosis but also hold promise as biomarkers for early HCC diagnosis. Flow cytometric analysis of peripheral blood Tfh and follicular regulatory T cells (Tfr cells) in 110 HCC patients revealed that the Tfr-Tfh index (TTI) demonstrates favourable diagnostic efficacy for early HCC and recurrence status [142].

Multiple studies have also demonstrated that the level of follicular helper T cell infiltration is a risk factor influencing survival rates and prognosis in patients with hepatocellular carcinoma, with high follicular helper T cell infiltration significantly associated with poorer overall survival. Research has indicated that tissue-resident Tfh-cell-like cells in hepatocellular carcinoma operate via the IL-21-IFNγ pathway, inducing plasma cells and creating conditions conducive to M2b macrophage polarisation [143].

## 5. Th Cell Subset Interactions in Hepatocellular Carcinoma Development and Treatment

### 5.1. The Role of T Helper Cells in the Progression from MASH to HCC

During the progression from MASH to HCC, significant remodelling of the hepatic immune microenvironment occurs. Previous research employing spatial proteomics techniques has demonstrated that the tumour microenvironment transitions from a pro-inflammatory state to an immunosuppressive one [144].

In the early inflammatory phase, the initial stage of the disease is marked by increased CD4^+^ T cell infiltration, with a tendency toward pro-inflammatory subset polarisation. Th1 cells activate dendritic cells by secreting IFN-γ and TNF-α and amplifying inflammatory signals. TFF2 secreted by hepatocytes has been shown to synergise with CXCL12 to promote the differentiation of CD4^+^ T cells toward Th1 and Th17 subsets, further exacerbating the inflammatory response [145,146]. CD4^+^ T cells may shift from the typical Th1 core cellular immune response towards a Th17 orientation [147]. Th17 cells play a pivotal role in the initiation, development, and progression of hepatocellular carcinoma. At this stage, Th17 cells emerge as the most abundant immune cell subset, secreting large quantities of IL-17. Their numbers rise significantly with the progression of metabolic inflammation, especially during the transition from MASH to liver fibrosis [148,149,150]. Particularly in patients with combined liver fibrosis, enhanced glycolytic metabolism and pro-inflammatory functions are demonstrated. Hepatic vascular dysregulation has been shown to enrich pro-fibrotic Th17 cells and accelerate the progression of liver fibrosis [151,152].

As the disease progresses towards HCC, the immune microenvironment undergoes a qualitative transformation. Metabolic dysfunction-associated steatohepatitis leads to the selective loss of CD4(^+^) T lymphocytes, thereby promoting the development of hepatocellular carcinoma [153]. Tregs have been shown to undergo significant expansion and functional conversion, with increased numbers and a high expression of amphiregulin (Areg). This has been shown to directly activate hepatic stellate cells and promote fibrosis. Meanwhile, a subset of Treg cells express IL-17, which correlates with more severe hepatic lesions [154]. The reduction in the effector subset and increase in the immunosuppressive subset signify a shift in the tumour microenvironment from a Th1/Th17-driven inflammatory state to a Treg-dominant immunosuppressive state. This leads to the loss of immune surveillance function, ultimately promoting the progression of hepatocellular carcinoma [155,156].

It is worth noting that during this process, Th22 cells exert a protective effect by secreting IL-22, thereby attenuating hepatocyte injury and inflammatory responses [157,158]. This suggests that Th22 cells may possess potential anti-tumour effects in the progression of MASH-HCC.

### 5.2. Th1/Th2 Imbalance in Hepatocellular Carcinoma Progression and Immune Evasion

**Figure 4:**
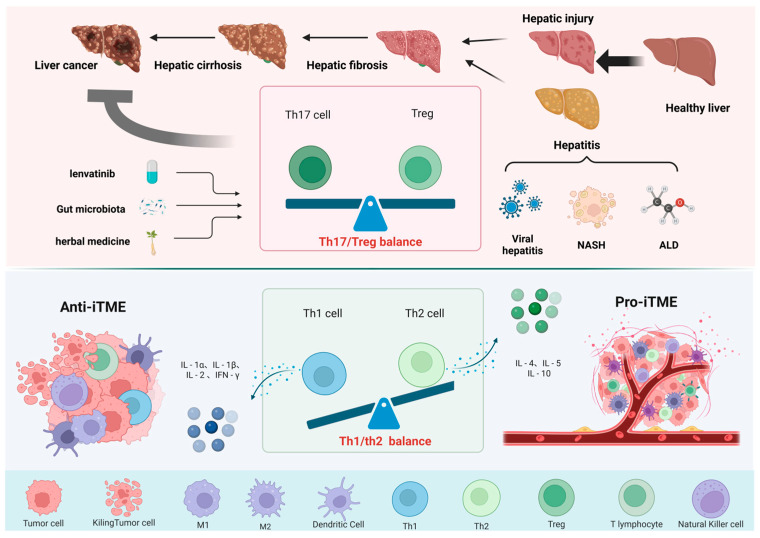
Changes in the balance of immune cells during the progression of liver cancer. The upper section illustrates the pathological progression from a healthy liver to hepatitis, hepatic fibrosis, cirrhosis, and hepatocellular carcinoma, with alterations in the Th17/Treg balance playing a pivotal role in disease advancement [167,168]. The lower section demonstrates the impact of Th1/Th2 balance on the tumour microenvironment, where anti-tumour immune cells and pro-tumour immune cells regulate tumour growth and immune evasion through the secretion of distinct cytokines [159,160].

Th1 and Th2 cells, as two crucial subsets of Th cells, maintain a relative equilibrium by secreting cytokines that mutually inhibit each other’s immune responses. However, within the tumour microenvironment, alterations in cytokine types or concentrations may disrupt this balance, prompting Th1 and Th2 cells to undergo transformation (Figure 4).

Research indicates that the shift from Th1 to Th2 cytokines serves as a crucial marker in the establishment of an immunosuppressive tumour microenvironment. Elevated levels of Th1 cytokines (IL-1α, IL-1β, IL-2, and IFN-γ) in tumour tissues, as revealed by gene expression microarray analysis, are often associated with a more favourable prognosis. In contrast, increased expression of Th2 cytokines (IL-4, IL-5, and IL-10) is commonly observed in HCC cases, as shown by similar genomic profiling techniques [159]. In patients with liver cancer, Th1 cell numbers decrease and their function weakens, while Th2 cell numbers increase, and their function strengthens. This imbalance in the Th1/Th2 ratio promotes tumour metastasis [160]. In a mouse model of hepatocellular carcinoma, shifting the Th1/Th2 balance towards Th1 dominance effectively alleviates the immunosuppressive tumour microenvironment [161]. He, X. et al. discovered that by inducing downregulation of Tregs in H22 hepatocellular carcinoma-bearing mice via glycyrrhizin polysaccharides, and subsequently increasing the Th1/Th2 cytokine ratio in serum, tumour growth in these mice could be inhibited [162]. Similarly, Jia et al. discovered that restoring the Th1/Th2 immune balance—specifically by upregulating pro-inflammatory cytokines IFN-γ and IL-2 while downregulating anti-inflammatory cytokines IL-4 and IL-10—exerted therapeutic effects on hepatocellular carcinoma-induced splenomegaly in mouse models, thereby alleviating symptoms of fatigue in liver cancer mice [163].

Moreover, the Th1/Th2 balance influences both the treatment and prognosis of liver cancer. In hepatocellular carcinoma, the macrophage colony-stimulating factor (CSF-1) modulates tumour-associated macrophages (TAMs), which regulate the tumour microenvironment, thereby affecting the equilibrium between Th1 and Th2 cells. When TAMs are reduced, the Th1/Th2 cytokine balance is enhanced, thereby improving the efficacy of immune checkpoint inhibitor therapy for HCC. Further studies reveal that increasing Th1 expression indirectly lowers PD-1/PD-L1 expression, significantly reducing tumour growth [164,165]. Research indicates that a higher Th1/Th2 ratio correlates with a more robust anti-tumour immune response, reduced tumour cell proliferation, and improved overall survival [166].

In summary, understanding the mechanisms of Th1/Th2 regulation in hepatocellular carcinoma holds significant theoretical and practical implications for developing novel therapeutic approaches and improving patient outcomes.

### 5.3. Th17/Tregs Imbalance Promotes the Progression of Hepatocellular Carcinoma

An imbalance between Th17 and Treg cells has been identified as a significant risk factor for the development of hepatocellular carcinoma, with studies showing that this imbalance is associated with the liver (Figure 4). The density of FOXP3^+^ Tregs infiltrating the liver progressively increases from chronic hepatitis B (CHB) to acute hepatitis (AH) and ultimately to HCC. Conversely, the densities of IL-17 and CD8^+^ T cells show a decreasing trend. The Th17/Tregs ratio undergoes significant alterations, closely correlating with the progressive decline in liver function. This confirms the existence of a delicate equilibrium between Th17 and Treg cells within the liver [167,168]. In this process, IL-6 and TGF-β play a pivotal role in regulating the equilibrium between Th17 and Tregs. Research indicates that TGF-β promotes the differentiation of Th17 cells by inducing FoxP3^+^ Treg cells via IL-6. [169,170]. At low concentrations of TGF-β, IL-6 and IL-21 act synergistically to promote IL-23R expression, thereby further driving Th17 differentiation. Conversely, under high TGF-β concentrations, the expression of Th17 cell hallmarks—such as IL-23R and IL-22—is suppressed. Concurrently, increased Foxp3 inhibits RORγt activity, subsequently promoting the differentiation of iTreg cells [171]. This indicates that IL-6 may not only promote the conversion of nTregs into Th17 cells but may also play a role in the differentiation of other Th cells [172].This study further revealed that HIF-1α in the liver regulates Th17 differentiation via IL-6 and influences Treg expansion under hypoxic conditions, thereby further affecting the hepatic immune microenvironment [173].

The most common precipitating factor for hepatocellular carcinoma is hepatitis, which plays a significant role in its actual development. An imbalance between Tregs and Th17 cells may also promote hepatocellular carcinoma through contributing factors such as viral hepatitis and alcoholic liver damage, further increasing the likelihood of its occurrence. Liu, B et al. analysed 38 patients with low-grade or moderate chronic hepatitis B (CHB-LM), 20 patients with chronic severe hepatitis B (CSHB), and 10 healthy controls (HCs). Their findings revealed that an imbalance in Th17/Tregs correlates with chronic HBV infection and liver damage [174,175]. An imbalance between Th17 and Treg cells is also regarded as a risk factor for developing hepatocellular carcinoma in patients with HBV infection [176]. Research has shown that an imbalance between Tregs and Th17 cells is associated with the promotion of hepatocellular carcinoma, especially in the context of chronic hepatitis B and other liver diseases [177]. Moreover, in mouse model studies, it has been observed that an imbalance between Th17 and Treg cells in the liver occurs concurrently with hepatic injury. Neutralising IL-17A can mitigate the extent of liver damage and also improve neutrophil infiltration. Furthermore, this hepatic imbalance in Th17/Tregs exacerbates LPS-induced liver injury [178]. Research has revealed that an imbalance in the Th17/Tregs ratio leads to the death of normal hepatocytes through ferroptosis, constituting one of the risk factors for MASH This imbalance promotes the development and progression of MASH [179,180]. Furthermore, Liu, J et al. found that chronic intermittent hypoxia exacerbates this imbalance by promoting Th17 differentiation and suppressing Treg expansion, thereby accelerating the progression of non-alcoholic steatohepatitis [181]. The equilibrium between Th17 and Treg cells maintains immune function. Persistent disruption of this balance triggers systemic inflammation, leading to immune system dysfunction and marked deterioration in liver function and blood lipid levels. This aligns with changes in relevant indicators observed in non-alcoholic fatty liver disease rats in existing research reports [182]. Based on the preceding research discussions, disruption of the balance between Tregs and Th17 cells promotes the development and progression of MASH. The imbalance between Th17 and Treg cells remains a significant indicator of the progression of liver cirrhosis. K. Li et al. observed that patients with hepatitis B-related liver cirrhosis exhibited an imbalance in Tregs/Th17 ratios. This included a reduction in peripheral blood Tregs and an increase in Th17 cells, resulting in a diminished Tregs/Th17 ratio. Furthermore, a negative correlation was observed between Tregs and Th17 cell counts. This ratio imbalance correlates closely with the clinical staging of hepatitis B-related cirrhosis, establishing the Tregs/Th17 ratio as a favourable biomarker for predicting decompensated cirrhosis [176,183].

In HCC-related studies, it was observed that the proportion of Treg cells in the HCC group was significantly higher than in the non-tumour control group. Concurrently, the proportion of Th17 cells in the HCC group was also markedly elevated compared to the non-tumour control group. Similarly, the Tregs/Th17 ratio in the HCC group was significantly increased relative to the non-tumour control group [184]. This indicates an imbalance between Treg and Th17 cells in HCC patients, with disrupted Th17/Treg expression levels associated with hepatocellular carcinoma. Furthermore, this imbalance contributes to the pathogenesis of HCC.

### 5.4. Improving the Th/Treg Cell Balance to Inhibit Liver Cancer Progression

In studies investigating Th17/Treg-based therapies for liver cancer, multiple investigations identified several effective treatment strategies. Mutually beneficial microorganisms from the gut microbiota inhabiting the gastrointestinal tract play a vital role in digestion, immunity, and cancer prevention. Alterations in its microenvironment can trigger inflammation-associated cancers [185]. Research indicates that the gut microbiota can promote immune homeostasis in both the intestine and extraintestinal organs (lung, liver, brain, kidney, and bone) by balancing Th17/Treg cell populations [186]. For instance, a combination of bifidobacteria, lactobacilli and enterococci probiotics can restore gut microbial balance, thereby enhancing the efficacy of conventional treatments for autoimmune hepatitis (AIH). This mechanism of action involves suppressing the imbalance of Th17/Tregs in the liver by inhibiting IL-33 upregulation mediated through the TLR2/4 signalling pathway [187]. Furthermore, Lactobacillus rhamnosus GG exhibits distinct advantages by enhancing intestinal barrier function. It effectively reverses the reduction in Tregs and increased IL-17 secretion by Th17 cells in peripheral blood caused by alcohol exposure, thereby mitigating alcohol-induced liver damage [188,189]. Similarly, Clostridium butyricum (B1) plays a significant role as it regulates the balance of Th17/Treg cells within the liver and gut, demonstrating a positive effect in alleviating high-fat diet (HFD)-induced fatty liver inflammation in mice [190]. Huo, R et al. discovered that stigmasterol influences Lactobacillus johnsonii, Lactobacillus murinus and Lactobacillus reuteri, leading to increased proportions of IFN-γ^+^CD8^+^ T cells and Tregs in both intestinal mucosa and tumour tissues. This reshapes the gut microbiota, thereby inhibiting tumour growth in hepatocellular carcinoma [191]. These studies indicate that the gut microbiota can mitigate liver inflammation and injury by regulating the Th17/Tregs balance, thereby inhibiting the occurrence and progression of hepatocellular carcinoma.

Herbal medicine, as a traditional therapeutic modality, has demonstrated extensive potent anti-cancer properties, particularly in enhancing tumour immune responses. Chinese herbal formulas offer differentiated intervention strategies by multi-dimensionally regulating the equilibrium of T cell subsets. It exhibits unique advantages, especially in reversing the imbalance between Tregs and effector Th cells [192]. Da Huang Zhe Chong Wan (DHZCP), as a representative example, exhibits significant tumour-suppressing effects in a mouse HCC model [193]. Its core mechanism involves reversing the Tregs/Th1 balance: post-treatment, the proportion of Th1 cells in the peripheral blood and spleen increases markedly, accompanied by heightened IFN-γ secretion, thereby activating CTLs. Conversely, Treg generation was suppressed, which synergistically reduced tumour volume and weight [194]. This pattern of restoring immune surveillance through immune equilibrium regulation exemplifies the multi-targeted nature of TCM formulas—they do not directly kill tumours but instead reshape anti-tumour immune responses by precisely modulating the differentiation and polarisation of Th cells.

Similar immunomodulatory mechanisms are observed in other spleen-tonifying and heat-clearing formulas. The spleen-tonifying and Stasis-Resolving Formula (JHD) effectively alleviates immunosuppression in the H22 hepatocellular carcinoma model by reversing imbalances in splenic T-lymphocyte subsets in HCC mice. This is achieved by increasing the proportion of CTLs while decreasing Treg and Th17 cells [195]. Research indicates that Scutellaria baicalensis extract successfully inhibits tumour growth in the H22 mouse hepatocellular carcinoma model by downregulating Treg numbers and modulating Th1/Th17 immune responses [196]. Glycyrrhiza root polysaccharides (GPs) reduced the proportion of Treg cells, decreased Foxp3 expression within Tregs, and upregulated the Th1/Th2 cytokine ratio in serum from tumour-bearing mice, potentially explaining part of the tumour growth inhibition effect [162].

Concurrently, N-glycosylated LTβR enhances the Th17/Tregs cell ratio by stabilising RORC and suppressing FOXP3, thereby amplifying local anti-tumour immunity and inhibiting HCC progression [197]. Research by Ren, H. et al. has also demonstrated that reversing the imbalance between Th17 and Treg cells can effectively mitigate the recurrence of liver cancer induced by hepatic ischaemia–reperfusion injury [126]. Lenvatinib is a multi-targeted tyrosine kinase inhibitor known to inhibit tumour angiogenesis and proliferation by blocking signalling pathways, including vascular endothelial growth factor receptors, fibroblast growth factor receptors, and platelet-derived growth factor receptors [198]. Following lenvatinib treatment for hepatocellular carcinoma, the frequency of Th cells and Tregs decreased, whilst the frequency of CTLs significantly increased [72], thereby improving the immune status of liver cancer patients, preventing effector cells from becoming exhausted, and suppressing the number and function of immunosuppressive cells.

## 6. Therapeutic Strategies of T-Helper Cells in Hepatocellular Carcinoma

Due to the global ageing population, the incidence and mortality rates of liver cancer are on the rise. Presently, no drug therapy exists that can completely halt or reverse the progression of liver cancer. Therefore, identifying effective therapeutic approaches is imperative. Although current treatments for hepatocellular carcinoma primarily involve surgical resection, targeted therapy, and immune checkpoint inhibitors, these methods offer only partial efficacy. However, they cannot entirely halt disease progression, may fail to adequately address all manifestations of the disease, particularly in patients at an advanced stage of disease, and may induce adverse side effects. Given the critical role of the immune microenvironment in the pathological development of hepatocellular carcinoma, immunotherapy has emerged as a potential avenue for treating this disease.

### 6.1. Targeted Therapy for Suppression of Tregs in Hepatocellular Carcinoma

Within the immunological microenvironment of hepatocellular carcinoma, Tregs constitute a pivotal subset that maintains immune tolerance and drives tumour immune evasion. Recent years have witnessed systematic elucidation of therapeutic strategies targeting Tregs across multiple dimensions, including phenotypic reprogramming, vaccine intervention, molecular regulation, and natural medicines (Table 1). Regarding phenotypic reprogramming, the CCR8 antagonist IPG0521m reverses tumour-infiltrating CCR8^+^ Tregs to a low-suppressive phenotype [199]. The fully human CD137 antibody P1A1 significantly suppresses the progression of mouse HCC by depleting CD137^+^ Tregs [200]. In terms of vaccine strategy, virus-like silicon nanoparticles (V-scVLPs) with a spike-like topology can co-deliver HCC neoantigens and TLR9 agonists, thereby downregulating TIM-3 on CD8^+^ T cells and reducing Treg infiltration. This approach lowers the proportion of regulatory T cells while increasing cytokine levels, thereby altering the tumour microenvironment to effectively suppress established in situ HCC tumour growth [201]. At the molecular regulatory level, miR-15a/16-1 can block cross-talk between Kupffer cells and Tregs [202], and miR-206 disrupts the synergistic interaction between c-Myc^+^ malignant hepatocytes and Tregs, thereby weakening the immunosuppressive network twofold [203]. In terms of signal transduction, Traf6 inhibitors suppress multiple signalling pathways, impeding the migration and reducing the number of Tregs infiltrating tumours. This prevents the suppression of T cell-mediated anti-tumour immunity, thereby enhancing the anti-tumour immune response [204]. Natural small molecules also enhance therapeutic approaches: resveratrol selectively downregulates CD8^+^CD122^+^ regulatory T cells [205], and HBE nanocrystal formulations (HBE NCs) promote dendritic cell maturation, synergistically depleting Tregs and myeloid-derived suppressor cells (MDSCs), thereby achieving a “cold-to-hot” tumour phenotype conversion [206]. In summary, multi-modal, multi-targeted interventions targeting Tregs have emerged as a significant approach for enhancing immune response in hepatocellular carcinoma therapy.

### 6.2. Therapeutic Approaches for Enhancing Th1 Responses in Hepatocellular Carcinoma

In research into therapeutic strategies for hepatocellular carcinoma, multiple approaches have demonstrated potential for inhibiting tumour growth by enhancing Th1 responses (Table 2). Research indicates that the long non-coding RNA (lncRNA) MEG3 significantly amplifies Th1 responses by stimulating M1-type macrophage polarisation and suppressing colony-stimulating factor 1 (CSF-1) expression. Its overexpression (OE) leads to elevated M1 marker expression, substantial increases in Th1 cytokines, and concurrently reduces PD-1/PD-L expression levels on macrophages [164]. Similarly, prophylactic and therapeutic vaccines developed from live attenuated monocyte-expanded bacteria effectively induce specific Th1 immune responses, leading to a significant increase in tumour-specific IFN-γ-producing CD4 and CD8 T cells. Prophylactic vaccination with the LmAIO vaccine not only reduces the incidence of HCC but also induces a pronounced tumour-specific Th1 immune response, thereby lowering tumour-specific IgG levels [208]. Furthermore, glycyrrhetinic acid (GA)-modified biphenyl mustard prodrugs have demonstrated significant efficacy in treating hepatocellular carcinoma. This is achieved by enhancing the ratio of CD4^+^ T cells to CD8^+^ T cells at the tumour site, promoting the differentiation of CD4^+^ T cells towards Th1 cells, and reducing the proportion of Tregs and Th2 cell subsets. Concurrently, these agents interfere with tumour cell DNA replication processes and modulate the tumour microenvironment [209]. The application of the novel ionisable lipid FS01-LNP has enhanced the transfection efficiency, immunogenicity, and safety profile of lipid nanoparticles. This lipid induces potent antigen-specific antibodies, memory B cells, and Th1-biassed T cell responses whilst demonstrating excellent balanced innate immune activation with minimal inflammatory response and hepatotoxicity [210]. Zol-treated HCC cell lines can trigger γδ T cell proliferation and induce the production of Th1 and Th2 cytokines, but do not induce Th17 cytokine production [211]. In terms of nanoparticles, the engineered mature core–shell nanoparticles (GHC NPs) loaded with GA, heparin (HP), and the immunostimulatory cytidine–phosphate–guanine oligonucleotide (CpG ODN) demonstrated significant inhibitory effects on tumour cell proliferation and angiogenesis. Furthermore, they induced a Th1 immune response, thereby restricting tumour growth [212]. These findings collectively offer diverse potential strategies for immunotherapy in hepatocellular carcinoma. By modulating Th1 cell-related immune responses, they enhance the body’s capacity for tumour immune surveillance and clearance, presenting broad prospects for future clinical application.

### 6.3. Targeted Therapy for Suppression of Th17 Cells in Hepatocellular Carcinoma

Within the HCC microenvironment, Th17 cells form an “inflammation–tumour” positive feedback loop through sustained IL-17A secretion. Th17 cells have emerged as a key subpopulation promoting tumour growth and metastasis. Dual-targeted intervention at both the differentiation and functional stages has demonstrated clear therapeutic potential (Table 3). At the upstream differentiation level, the transcription factor ROR-γt is regarded as the “master switch”. Metformin directly inhibits CD3/CD28-induced ROR-γt expression and blocks STAT3 phosphorylation along with the transcription of its downstream targets Bcl-2 and Cyclin D1, thereby significantly reducing the tumour burden in orthotopic mouse models [213]. Concurrently, N-glycosylated LTβR reduces the Th17/Treg cell ratio by inhibiting the ubiquitin-mediated degradation of RORC (encoded by the ROR-γt gene), thereby further attenuating its tumour-promoting effects [197]. At the downstream effector level, the IL-17A/IL-17RA signalling axis has emerged as a precise intervention target. To date, three IL-17A/IL-17RA inhibitors have received regulatory approval: secukinumab, ixekizumab, and brodalimumab [214]. Among these, Scythemonab demonstrated particularly outstanding efficacy in HCC models. When combined with IL-35, it inhibits Notch signalling via the Snail/E-cadherin axis, significantly attenuating tumour cell invasion [215]. When combined with starvation therapy, it further restricts tumour cell survival by downregulating BCL2 and inducing autophagic death [216]. Combination therapy with sorafenib demonstrated superior tumour growth inhibition and control of intrahepatic metastases compared to monotherapy [117]. Moreover, a novel probiotic mixture termed Prohep can reduce the activity of Th17 cells—the primary producers—by downregulating IL-17 cytokine levels. This subsequently diminishes the frequency of Th17 cells in both the gut and peripheral blood, thereby significantly slowing the progression of hepatocellular carcinoma [217]. In summary, the dual-lock strategy of targeting ROR-γt to block Th17 differentiation and neutralising IL-17A/IL-17RA to counteract its effectors offers a rapidly clinically translatable combination approach for remodelling the HCC immune microenvironment.

**Table 3:** Overview of hepatocellular carcinoma treatment strategies targeting Th17 cell suppression.

Treatment Methods	Treatment Strategy	Evidence Level	Efficacy Indicators	References
Secukinumab and sorafenib	Blocking IL-17A	In vivo (mouse models of HCC)	Reduced BCL2 protein expression and autophagy suppression.	[117]
Secukinumab and starvation therapy	Blocking IL-17A	In vivo (mouse models of HCC)	Suppressed tumour growth, reduced IL-17A secretion, and enhanced autophagy	[216]
Metformin	Inhibition of ROR-γt expression	In vivo (mouse models of HCC)	Tumour growth inhibition, decreased IL-22 secretion, and suppressed Th1/Th17 differentiation.	[213]
Probiotic blend	Regulating Gut Microbiota	In vivo (mouse models of HCC)	40% tumour growth reduction, enhanced gut microbiota, reduced Th17 recruitment.	[217]

### 6.4. Targeted T-Helper Cell-Based Immunotherapy Clinical Trials for Liver Diseases

Multiple ongoing clinical trials are investigating the use of various immunotherapies across different liver-related diseases (Table 4), particularly in the treatment of HCC, chronic hepatitis C, and liver transplantation. These trials focus on modulating Th cell subsets, specifically Th1, Th17, and Tregs, to enhance the immune system’s response to tumours [218,219,220]. Through this approach, immunotherapy holds promise for improving the liver’s immune microenvironment, promoting anti-tumour immune responses and simultaneously suppressing immune escape mechanisms, thereby playing a significant role in treating liver-related diseases.

## 7. Conclusions and Outlook

The development of HCC is closely associated with hepatitis, chronic liver injury, hepatic fibrosis, and immune tolerance regulation. Th cells, as a vital component of the adaptive immune system, play a pivotal role in tumour immune responses, immune evasion, and therapeutic outcomes. Imbalances among Th cell subsets drive tumour progression and limit the efficacy of monotherapies targeting single immune checkpoints.

In recent years, immune checkpoint inhibitors (ICIs), particularly monoclonal antibodies targeting PD-1/PD-L1 and CTLA-4, have become the new standard of care for advanced HCC. Clinical trials such as IMbrave 150 and HIMALAYA demonstrate that these therapies significantly improve OS and progression-free survival in patients. However, objective response rates remain below 30%, revealing the complexity of the tumour-suppressive microenvironment, which remains a major therapeutic challenge [66,221].

Beyond ICIs, novel immunotherapies, including CAR-T cell therapy, cancer vaccines, and oncolytic viruses, are emerging as frontiers in HCC treatment. Th1-based vaccines have already entered clinical trials. By activating Th1 cell responses, these immunotherapies demonstrate clinical potential to enhance immune responses and improve treatment outcomes, offering hope for more durable therapeutic effects in HCC patients [219].

However, these immunotherapeutic interventions also carry potential risks. Specifically, manipulations inducing inflammatory Th subsets (such as Treg and Th17 cells) may disrupt immune homeostasis, leading to immune overactivation. This could increase the risk of autoimmune diseases and even potentially promote tumourigenesis. Furthermore, the gut microbiota, as a crucial regulator of the hepatic immune system, has garnered increasing attention in recent years. The gut–liver axis facilitates interactions between the gut microbiota and the hepatic immune system, modulating immune responses. Dysbiosis of the gut microbiota is closely associated with the development and progression of HCC. Improving microbial composition may restore immune equilibrium, enhance anti-tumour immune responses, and mitigate the side effects of immunotherapy, offering novel therapeutic approaches.

In summary, while immunotherapy—particularly ICIs and novel immunotherapies—has demonstrated significant potential in HCC treatment, the complexity of the immune microenvironment and its potential immunopathological consequences remain therapeutic challenges. Future research should explore strategies to optimise regulatory T cell modulation, balance immune responses, reduce adverse effects, and enhance the regulatory role of the gut microbiota, thereby achieving more effective and durable HCC treatment.

## Figures and Tables

**Table 1:** Overview of hepatocellular carcinoma treatment strategies targeting Tregs.

Treatment Methods	Treatment Strategy	Evidence Level	Efficacy Indicators	References
CCR4 antagonist	Targeting CCR4^+^ Tregs	In vivo (mouse models of HCC)	Inhibited tumour growth, enhanced immunity, reduced tumour weight, and improved survival. PD-1 blockade boosted CD8^+^ T cell function.	[207]
CCR8 antagonist	Targeting the CCR8 Receptor	In vivo (mouse models of HCC)	Inhibited tumour growth, increased CD8^+^ T cell infiltration, and reduced Treg immunosuppression.	[199]
microRNA(15a/16-1)	Targeting Kupffer Cells and Tregs	In vivo (mouse models of HCC)	Prevented HCC, reduced hepatic Tregs, increased CTL and CD8^+^ activity, and disrupted NF-kB-CCL22 signalling.	[202]
microRNA(206)	Blocking miR-206-Kras Interaction	In vivo (c-Myc-driven mouse models)	Reduced TGF-β production, restored liver Treg/CD8^+^ ratio, and promoted HCC regression.	[203]
Cancer vaccine	Co-delivery of Neoantigens and TLR9 Agonists	In vivo (mouse orthotopic HCC models)	Tumour growth inhibition, enhanced CD8^+^ T cell activation, increased central memory T cell responses, reduced Tregs, and improved tumour microenvironment.	[201]
IgG monoclonal antibody (P1A1)	Targeting CD137	In vivo (mouse models of HCC)	Tumour growth inhibition, reduced Tregs infiltration, and enhanced NK cell-mediated cytotoxicity.	[200]
Traf6 inhibitor	Inhibition of Traf6 Activity	In vivo (Hepa1-6 murine liver cancer models)	“Reduced tumour growth, inhibited CD8^+^ CD122^+^ Treg generation, and enhanced CD8^+^ T cell activity.”	[204]
Resveratrol	Targeting CD8^+^CD122^+^ Regulatory T Cells	In vivo (Hepa1-6 and H22 murine models)	Reduced tumour growth, inhibited CD8^+^ CD122^+^ Treg generation, and enhanced CD8^+^ T cell cytotoxicity.	[205]
HBE NCs	Induction of Immunogenic Cell Death	In vivo (mouse models of HCC)	“Induced ICD, enhanced dendritic cell maturation, increased CD4^+^ and CD8^+^ T cell infiltration, and reduced Tregs and MDSCs.”	[206]

**Table 2:** Overview of hepatocellular carcinoma treatment strategies inducing Th1 cells.

Treatment Methods	Treatment Strategy	Evidence Level	Efficacy Indicators	References
LncRNA (MEG3)	Macrophage M1 Polarisation and Regulation by CSF-1	In vivo and in vitro (HCC models)	Increases Th1 cytokines, reduces Th2 cytokines, and promotes M1 polarisation.	[164]
Bacterial vaccine	Targeted Immune Checkpoint Inhibitor	In vivo (mouse models of HCC and CCA)	Induces strong Th1 immune responses and reduces tumour burden and tumour-specific IgG levels.	[208]
Glycyrrhetinic acid	Regulation of Tumour Microenvironment	In vivo (mouse models of HCC)	Increased proportion of CD4^+^ T and CD8^+^ T cells at the tumour site, promoting CD4^+^ T cell differentiation into Th1 cells, and reducing Treg and Th2 cell subsets.	[209]
Lipid nanoparticles (FS01)	mRNA Delivery	In vivo (mouse models of HCC)	Robust antibody and memory B cell responses and enhanced Th1 immune responses.	[210]
Zoledronic acid	γδ T Cell Proliferation and Cytokine Production	In vivo (HCC cell line studies)	Enhances γδ T cell-mediated killing, increasing Th1 cytokines.	[211]
Core–shell nanoparticles (GHC NPs)	Tumour Angiogenesis Inhibition and Th1 Immune Response	In vivo (mouse models of HCC)	Promotes Th1 responses, CD4^+^ T cell differentiation into Th1, and increases IFN-γ secretion.	[212]

**Table 4:** Clinical Trials of T-helper cells Therapy for Liver Disease.

Number	Conditions	Phases	Study Status	Interventions	Primary Outcome Measures
NCT05528952	Hepatocellular Carcinoma	II	RECRUITING	UCPVax (Th1-type vaccine)	Objective response rate, including complete response
NCT05033522	Hepatocellular Carcinoma	II/III	RECRUITING	AlloStim (Activate Th1-like CD4^+^ T cells)	Overall survival and time from randomisation to death from any cause
NCT00968357	Chronic Hepatitis C	II	COMPLETED	SCV-07 (Stimulate Th1-type immune responses)	The safety of SCV-07 was assessed at 2 doses for monotherapy as well as its immunomodulatory effects
NCT02050646	Autoimmune Hepatitis	NA	COMPLETED	Low-salt diet	Changes in pathogenic TH17 cell production from baseline
NCT03511365	MAFLD	I/II	TERMINATED	Probiotic formulation VSL	Changes in IL-17 levels with probiotic administration, recorded as percent decrease over 8 weeks
NCT01624077	Liver Transplant	I	UNKNOWN	Regulatory T cells	Patient and graft survival one year post-transplantation
NCT03577431	Liver Transplant	I/II	ACTIVE_NOT_RECRUITING	arTreg-CSB	Incidence of rejection events
NCT02166177	End-Stage Liver Disease	I/II	COMPLETED	Autologous regulatory T cell product	Rate of dose-limiting toxicities (DLTs) and cellular rejection
NCT02474199	Liver Transplant	I/II	COMPLETED	darTregs	Investigation of treatment-related grade 3 or higher adverse events

## Data Availability

No new data were created or analysed in this study.

## Abbreviations

The following abbreviations are used in this manuscript:

HCC	Hepatocellular carcinoma
Tregs	T regulatory cells
Th cells	T-helper cells
ICCA	Intrahepatic cholangiocarcinoma
HBV	Hepatitis B virus
HCV	Hepatitis C virus
TME	Tumour microenvironment
HSCs	Haematopoietic stem cells
LPCs	Lymphoid progenitor cells
DN	Double-negative thymocytes
DP	Double-positive thymocytes
MHC	Major histocompatibility complex
SP	Single-positive thymocytes
TCR	T cell receptor
APC	Antigen-presenting cell
IFN-γ	Interferon-γ
FOXP3	Forkhead Box P3
TIC	Tumour-initiating cell
EMT	Epithelial-to-mesenchymal transition
VEGF	Vascular endothelial growth factor
TACE	Transarterial chemoembolisation
SBRT	Stereotactic body radiotherapy
CTLs	Cytotoxic T lymphocytes
PHC	Primary hepatocellular carcinoma
OS	Overall survival
DFS	Disease-free survival
TLS	Tertiary lymphoid structures
CSF-1	Colony-stimulating factor-1
TAMs	Tumour-associated macrophages
MASH	Metabolic-associated steatohepatitis

## References

[1] Bray F., Laversanne M., Sung H., Ferlay J., Siegel R.L., Soerjomataram I., Jemal A. (2024). Global cancer statistics 2022: GLOBOCAN estimates of incidence and mortality worldwide for 36 cancers in 185 countries. CA Cancer J. Clin..

[2] Vogel A., Meyer T., Sapisochin G., Salem R., Saborowski A. (2022). Hepatocellular carcinoma. Lancet.

[3] Zhou J., Sun H., Wang Z., Cong W., Zeng M., Zhou W., Bie P., Liu L., Wen T., Kuang M. (2023). Guidelines for the Diagnosis and Treatment of Primary Liver Cancer (2022 Edition). Liver Cancer.

[4] Zhang X., Zhu F., Wen J., Pan Z., Zhu Y. (2025). Factors influencing the timing of second-line therapy for the treatment of advanced hepatocellular carcinoma: A systematic review and meta-analysis. Holist. Integr. Oncol..

[5] Akinyemiju T., Abera S., Ahmed M., Alam N., Alemayohu M.A., Allen C., Al-Raddadi R., Alvis-Guzman N., Amoako Y., Artaman A. (2017). The Burden of Primary Liver Cancer and Underlying Etiologies From 1990 to 2015 at the Global, Regional, and National Level: Results from the Global Burden of Disease Study 2015. JAMA Oncol..

[6] Yu L.X., Ling Y., Wang H.Y. (2018). Role of nonresolving inflammation in hepatocellular carcinoma development and progression. NPJ Precis. Oncol..

[7] Huang M., Wang D., Huang J., Bae A.N., Xia Y., Zhao X., Mortaja M., Azin M., Collier M.R., Semenov Y.R. (2025). Hepatitis B virus promotes liver cancer by modulating the immune response to environmental carcinogens. Nat. Commun..

[8] Bu L., Chen M., Liu S., Li T., Huang Z. (2025). A longitudinal study on symptom distress and management of transhepatic arterial interventional chemotherapy combined with targeted therapy and immunotherapy based on patient self-reported outcomes. Holist. Integr. Oncol..

[9] Tabrizian P., Jibara G., Shrager B., Schwartz M., Roayaie S. (2015). Recurrence of hepatocellular cancer after resection: Patterns, treatments, and prognosis. Ann. Surg..

[10] Yang J.D., Hainaut P., Gores G.J., Amadou A., Plymoth A., Roberts L.R. (2019). A global view of hepatocellular carcinoma: Trends, risk, prevention and management. Nat. Rev. Gastroenterol. Hepatol..

[11] Mirshahi F., Aqbi H.F., Isbell M., Manjili S.H., Guo C., Saneshaw M., Bandyopadhyay D., Dozmorov M., Khosla A., Wack K. (2022). Distinct hepatic immunological patterns are associated with the progression or inhibition of hepatocellular carcinoma. Cell Rep..

[12] Ringelhan M., Pfister D., O’Connor T., Pikarsky E., Heikenwalder M. (2018). The immunology of hepatocellular carcinoma. Nat. Immunol..

[13] Speiser D.E., Chijioke O., Schaeuble K., Münz C. (2023). CD4(+) T cells in cancer. Nat. Cancer.

[14] Saravia J., Chapman N.M., Chi H. (2019). Helper T cell differentiation. Cell Mol. Immunol..

[15] Bick F., Blanchetot C., Lambrecht B.N., Schuijs M.J. (2025). A reappraisal of IL-9 in inflammation and cancer. Mucosal Immunol..

[16] Kumar R., Theiss A.L., Venuprasad K. (2021). RORγt protein modifications and IL-17-mediated inflammation. Trends Immunol..

[17] Kang J.H., Zappasodi R. (2023). Modulating Treg stability to improve cancer immunotherapy. Trends Cancer.

[18] Fathi F., Saidi R.F., Banafshe H.R., Arbabi M., Lotfinia M., Motedayyen H. (2022). Changes in immune profile affect disease progression in hepatocellular carcinoma. Int. J. Immunopathol. Pharmacol..

[19] Borst J., Ahrends T., Bąbała N., Melief C.J.M., Kastenmüller W. (2018). CD4(+) T cell help in cancer immunology and immunotherapy. Nat. Rev. Immunol..

[20] Kumar B.V., Connors T.J., Farber D.L. (2018). Human T Cell Development, Localization, and Function Throughout Life. Immunity.

[21] Meitei H.T., Lal G. (2023). T cell receptor signaling in the differentiation and plasticity of CD4(+) T cells. Cytokine Growth Factor Rev..

[22] Ashby K.M., Hogquist K.A. (2024). A guide to thymic selection of T cells. Nat. Rev. Immunol..

[23] Varanasi S.K., Kumar S.V., Rouse B.T. (2020). Determinants of Tissue-Specific Metabolic Adaptation of T Cells. Cell Metab..

[24] Choi J.O., Seo Y., Hwang S.S. (2025). Guardians of silence: Transcriptional networks in T cell quiescence. Exp. Mol. Med..

[25] Sun X., Gu R., Bai J. (2024). Differentiation and regulation of CD4(+) T cell subsets in Parkinson’s disease. Cell Mol. Life Sci..

[26] Hwang J.R., Byeon Y., Kim D., Park S.G. (2020). Recent insights of T cell receptor-mediated signaling pathways for T cell activation and development. Exp. Mol. Med..

[27] Burke K.P., Chaudhri A., Freeman G.J., Sharpe A.H. (2024). The B7:CD28 family and friends: Unraveling coinhibitory interactions. Immunity.

[28] Shah K., Al-Haidari A., Sun J., Kazi J.U. (2021). T cell receptor (TCR) signaling in health and disease. Signal Transduct. Target. Ther..

[29] Hoefig K.P., Heissmeyer V. (2018). Posttranscriptional regulation of T helper cell fate decisions. J. Cell Biol..

[30] Jay A., Pondevida C.M., Vahedi G. (2025). The epigenetic landscape of fate decisions in T cells. Nat. Immunol..

[31] Fang D., Zhu J. (2017). Dynamic balance between master transcription factors determines the fates and functions of CD4 T cell and innate lymphoid cell subsets. J. Exp. Med..

[32] Angkasekwinai P., Dong C. (2021). IL-9-producing T cells: Potential players in allergy and cancer. Nat. Rev. Immunol..

[33] Eyerich S., Eyerich K., Pennino D., Carbone T., Nasorri F., Pallotta S., Cianfarani F., Odorisio T., Traidl-Hoffmann C., Behrendt H. (2009). Th22 cells represent a distinct human T cell subset involved in epidermal immunity and remodeling. J. Clin. Investig..

[34] Li F., Tian Z. (2013). The liver works as a school to educate regulatory immune cells. Cell Mol. Immunol..

[35] Lee W.Y., Kubes P. (2008). Leukocyte adhesion in the liver: Distinct adhesion paradigm from other organs. J. Hepatol..

[36] Muscate F., Woestemeier A., Gagliani N. (2021). Functional heterogeneity of CD4(+) T cells in liver inflammation. Semin. Immunopathol..

[37] Erkelens M.N., Mebius R.E. (2017). Retinoic Acid and Immune Homeostasis: A Balancing Act. Trends Immunol..

[38] Klugewitz K., Blumenthal-Barby F., Schrage A., Knolle P.A., Hamann A., Crispe I.N. (2002). Immunomodulatory effects of the liver: Deletion of activated CD4+ effector cells and suppression of IFN-gamma-producing cells after intravenous protein immunization. J. Immunol..

[39] Knolle P.A., Schmitt E., Jin S., Germann T., Duchmann R., Hegenbarth S., Gerken G., Lohse A.W. (1999). Induction of cytokine production in naive CD4(+) T cells by antigen-presenting murine liver sinusoidal endothelial cells but failure to induce differentiation toward Th1 cells. Gastroenterology.

[40] Venzin V., Beccaria C.G., Perucchini C., Delfino P., Bono E.B., Giustini L., Moalli F., Grillo M., Fumagalli V., Laura C. (2025). CD4(+) T cells license Kupffer cells to reverse CD8(+) T cell dysfunction induced by hepatocellular priming. Nat. Immunol..

[41] Boettler T., Kelsch L., Thimme R. (2025). Kupffer cells facilitate intrahepatic CD4 T cell help. Trends Immunol..

[42] Trujillo-Ochoa J.L., Kazemian M., Afzali B. (2023). The role of transcription factors in shaping regulatory T cell identity. Nat. Rev. Immunol..

[43] Alvisi G., Termanini A., Soldani C., Portale F., Carriero R., Pilipow K., Costa G., Polidoro M., Franceschini B., Malenica I. (2022). Multimodal single-cell profiling of intrahepatic cholangiocarcinoma defines hyperactivated Tregs as a potential therapeutic target. J. Hepatol..

[44] Nishikawa H., Koyama S. (2021). Mechanisms of regulatory T cell infiltration in tumors: Implications for innovative immune precision therapies. J. Immunother. Cancer.

[45] Ormandy L.A., Hillemann T., Wedemeyer H., Manns M.P., Greten T.F., Korangy F. (2005). Increased populations of regulatory T cells in peripheral blood of patients with hepatocellular carcinoma. Cancer Res..

[46] Fu J., Zhang Z., Zhou L., Qi Z., Xing S., Lv J., Shi J., Fu B., Liu Z., Zhang J.Y. (2013). Impairment of CD4+ cytotoxic T cells predicts poor survival and high recurrence rates in patients with hepatocellular carcinoma. Hepatology.

[47] Fu J., Xu D., Liu Z., Shi M., Zhao P., Fu B., Zhang Z., Yang H., Zhang H., Zhou C. (2007). Increased regulatory T cells correlate with CD8 T-cell impairment and poor survival in hepatocellular carcinoma patients. Gastroenterology.

[48] Galassi C., Chan T.A., Vitale I., Galluzzi L. (2024). The hallmarks of cancer immune evasion. Cancer Cell.

[49] Yang M., Song X., Zhang F., Li M., Chang W., Wang Z., Li M., Shan H., Li D. (2025). Spatial proteomic landscape of primary and relapsed hepatocellular carcinoma reveals immune escape characteristics in early relapse. Hepatology.

[50] Zheng C., Zheng L., Yoo J.K., Guo H., Zhang Y., Guo X., Kang B., Hu R., Huang J.Y., Zhang Q. (2017). Landscape of Infiltrating T Cells in Liver Cancer Revealed by Single-Cell Sequencing. Cell.

[51] Langhans B., Nischalke H.D., Krämer B., Dold L., Lutz P., Mohr R., Vogt A., Toma M., Eis-Hübinger A.M., Nattermann J. (2019). Role of regulatory T cells and checkpoint inhibition in hepatocellular carcinoma. Cancer Immunol. Immunother..

[52] Zhang Y., Li X., Chen H., Li J., Guo X., Fang Y., Chen L., Li K., Zhang Y., Kong F. (2025). Cancer Cell-Derived Exosomal miR-500a-3p Modulates Hepatic Stellate Cell Activation and the Immunosuppressive Microenvironment. Adv. Sci..

[53] Tong T., Gao W., Jian H., Yang R., Zhang J., Li K., Zeng P. (2025). The role and potential mechanisms of exosomes in the progression of hepatocellular carcinoma. Holist. Integr. Oncol..

[54] Park S., Hall M.N. (2025). Metabolic reprogramming in hepatocellular carcinoma: Mechanisms and therapeutic implications. Exp. Mol. Med..

[55] Chen L., Huang M. (2024). Oncometabolites in cancer: From cancer cells to the tumor microenvironment. Holist. Integr. Oncol..

[56] Gu J., Zhou J., Chen Q., Xu X., Gao J., Li X., Shao Q., Zhou B., Zhou H., Wei S. (2022). Tumor metabolite lactate promotes tumorigenesis by modulating MOESIN lactylation and enhancing TGF-β signaling in regulatory T cells. Cell Rep..

[57] Watson M.J., Vignali P.D.A., Mullett S.J., Overacre-Delgoffe A.E., Peralta R.M., Grebinoski S., Menk A.V., Rittenhouse N.L., DePeaux K., Whetstone R.D. (2021). Metabolic support of tumour-infiltrating regulatory T cells by lactic acid. Nature.

[58] Yasukawa K., Shimada S., Akiyama Y., Taniai T., Igarashi Y., Tsukihara S., Tanji Y., Umemura K., Kamachi A., Nara A. (2025). ACVR2A attenuation impacts lactate production and hyperglycolytic conditions attracting regulatory T cells in hepatocellular carcinoma. Cell Rep. Med..

[59] Wang Y., Chen W., Qiao S., Zou H., Yu X.J., Yang Y., Li Z., Wang J., Chen M.S., Xu J. (2024). Lipid droplet accumulation mediates macrophage survival and Treg recruitment via the CCL20/CCR6 axis in human hepatocellular carcinoma. Cell Mol. Immunol..

[60] Zhou X., Cui G., Hu E., Wang X., Tang D., Zhang X., Ma J., Li Y., Liu H., Peng Q. (2025). The impact of de novo lipogenesis on predicting survival and clinical therapy: An exploration based on a multigene prognostic model in hepatocellular carcinoma. J. Transl. Med..

[61] Chen F., Gong M., Weng D., Jin Z., Han G., Yang Z., Han J., Wang J. (2024). Phellinus linteus activates Treg cells via FAK to promote M2 macrophage polarization in hepatocellular carcinoma. Cancer Immunol. Immunother..

[62] Liu C., Tu Y.J., Cai H.Y., Pan Y.Y., Wu Y.Y., Zhang L. (2024). Regulatory T cells inhibit FoxP3 to increase the population of tumor initiating cells in hepatocellular carcinoma. J. Cancer Res. Clin. Oncol..

[63] Mempel T.R., Marangoni F. (2019). Guidance factors orchestrating regulatory T cell positioning in tissues during development, homeostasis, and response. Immunol. Rev..

[64] Facciabene A., Peng X., Hagemann I.S., Balint K., Barchetti A., Wang L.P., Gimotty P.A., Gilks C.B., Lal P., Zhang L. (2011). Tumour hypoxia promotes tolerance and angiogenesis via CCL28 and T(reg) cells. Nature.

[65] Hansen W., Hutzler M., Abel S., Alter C., Stockmann C., Kliche S., Albert J., Sparwasser T., Sakaguchi S., Westendorf A.M. (2012). Neuropilin 1 deficiency on CD4+Foxp3+ regulatory T cells impairs mouse melanoma growth. J. Exp. Med..

[66] Zhu A.X., Abbas A.R., de Galarreta M.R., Guan Y., Lu S., Koeppen H., Zhang W., Hsu C.H., He A.R., Ryoo B.Y. (2022). Molecular correlates of clinical response and resistance to atezolizumab in combination with bevacizumab in advanced hepatocellular carcinoma. Nat. Med..

[67] Sun Y., Wu L., Zhong Y., Zhou K., Hou Y., Wang Z., Zhang Z., Xie J., Wang C., Chen D. (2021). Single-cell landscape of the ecosystem in early-relapse hepatocellular carcinoma. Cell.

[68] Huang C.H., Ku W.T., Mahalingam J., Wu C.H., Wu T.H., Fan J.H., Su C.W., Lin P.T., Peng C.W., Yang C.K. (2025). Tumor-migrating peripheral Foxp3-high regulatory T cells drive poor prognosis in HCC. Hepatology.

[69] Liu F., Liu W., Sanin D.E., Jia G., Tian M., Wang H., Zhu B., Lu Y., Qiao T., Wang X. (2020). Heterogeneity of exhausted T cells in the tumor microenvironment is linked to patient survival following resection in hepatocellular carcinoma. Oncoimmunology.

[70] An Y., Gao S., Zhao W.C., Qiu B.A., Xia N.X., Zhang P.J., Fan Z.P. (2018). Transforming growth factor-β and peripheral regulatory cells are negatively correlated with the overall survival of hepatocellular carcinoma. World J. Gastroenterol..

[71] Sun H., Cao Z., Zhao B., Zhou D., Chen Z., Zhang B. (2025). An elevated percentage of CD4^+^CD25^+^CD127(low) regulatory T cells in peripheral blood indicates a poorer prognosis in hepatocellular carcinoma after curative hepatectomy. BMC Gastroenterol..

[72] Zhu J., Fang P., Wang C., Gu M., Pan B., Guo W., Yang X., Wang B. (2021). The immunomodulatory activity of lenvatinib prompts the survival of patients with advanced hepatocellular carcinoma. Cancer Med..

[73] Park H., Jung J.H., Jung M.K., Shin E.C., Ro S.W., Park J.H., Kim D.Y., Park J.Y., Han K.H. (2020). Effects of transarterial chemoembolization on regulatory T cell and its subpopulations in patients with hepatocellular carcinoma. Hepatol. Int..

[74] Zhao C.N., Chiang C.L., Chiu W.K., Chan S.K., Li C.J., Chen W.W., Zheng D.Y., Chen W.Q., Ji R., Lo C.M. (2025). Treatments of transarterial chemoembolization (TACE), stereotactic body radiotherapy (SBRT) and immunotherapy reshape the systemic tumor immune environment (STIE) in patients with unresectable hepatocellular carcinoma. J. Natl. Cancer Cent..

[75] Ren Z., Wang Y., Jiang D., Liu Y., Yang X., Wang T., Zhu J., Wang W., Chen Q., Zhang Y. (2025). PD1(+) Treg cell remodeling promotes immune homeostasis within peripheral blood and tumor microenvironment after microparticles-transarterial chemoembolization in hepatocellular carcinoma. Cancer Immunol. Immunother..

[76] Wu J., Zhou Z., Huang Y., Deng X., Zheng S., He S., Huang G., Hu B., Shi M., Liao W. (2024). Radiofrequency ablation: Mechanisms and clinical applications. MedComm.

[77] Behm B., Di Fazio P., Michl P., Neureiter D., Kemmerling R., Hahn E.G., Strobel D., Gress T., Schuppan D., Wissniowski T.T. (2016). Additive antitumour response to the rabbit VX2 hepatoma by combined radio frequency ablation and toll like receptor 9 stimulation. Gut.

[78] Liang J., Ma M., Feng W., Xu Q., Chen D., Lai J., Chen J. (2025). Anti-PD-L1 blockade facilitates antitumor effects of radiofrequency ablation by improving tumor immune microenvironment in hepatocellular carcinoma. Apoptosis.

[79] Zadka Ł., Grybowski D.J., Dzięgiel P. (2020). Modeling of the immune response in the pathogenesis of solid tumors and its prognostic significance. Cell Oncol..

[80] Coënon L., Geindreau M., Ghiringhelli F., Villalba M., Bruchard M. (2024). Natural Killer cells at the frontline in the fight against cancer. Cell Death Dis..

[81] Xu J., Ding L., Mei J., Hu Y., Kong X., Dai S., Bu T., Xiao Q., Ding K. (2025). Dual roles and therapeutic targeting of tumor-associated macrophages in tumor microenvironments. Signal Transduct. Target. Ther..

[82] Wu L., Deng H., Feng X., Xie D., Li Z., Chen J., Mo Z., Zhao Q., Hu Z., Yi S. (2024). Interferon-γ(+) Th1 activates intrahepatic resident memory T cells to promote HBsAg loss by inducing M1 macrophage polarization. J. Med. Virol..

[83] Dezhbord M., Kim S.H., Park S., Lee D.R., Kim N., Won J., Lee A.R., Kim D.S., Kim K.H. (2024). Novel role of MHC class II transactivator in hepatitis B virus replication and viral counteraction. Clin. Mol. Hepatol..

[84] Lee W.S., Yang H., Chon H.J., Kim C. (2020). Combination of anti-angiogenic therapy and immune checkpoint blockade normalizes vascular-immune crosstalk to potentiate cancer immunity. Exp. Mol. Med..

[85] Yu H., Lin G., Jiang J., Yao J., Pan Z., Xie H., Bo Z., He Q., Yang J., Chen Z. (2024). Synergistic activity of Enterococcus Faecium-induced ferroptosis via expansion of IFN-γ(+)CD8(+) T cell population in advanced hepatocellular carcinoma treated with sorafenib. Gut Microbes.

[86] Zhang N., Ye S., Wang X., Wang K., Zhong F., Yao F., Liu J., Huang B., Xu F., Wang X. (2024). Hepatic Symbiotic *Bacterium* L. reuteri FLRE5K1 Inhibits the Development and Progression of Hepatocellular Carcinoma via Activating the IFN-γ/CXCL10/CXCR3 Pathway. Probiotics Antimicrob. Proteins.

[87] Chang Y.S., Tu S.J., Chen H.D., Hsu M.H., Chen Y.C., Chao D.S., Chung C.C., Chou Y.P., Chang C.M., Lee Y.T. (2023). Integrated genomic analyses of hepatocellular carcinoma. Hepatol. Int..

[88] Behboudi S., Alisa A., Boswell S., Anastassiou J., Pathan A.A., Williams R. (2010). Expansion of anti-AFP Th1 and Tc1 responses in hepatocellular carcinoma occur in different stages of disease. Br. J. Cancer.

[89] Walker J.A., McKenzie A.N.J. (2018). T(H)2 cell development and function. Nat. Rev. Immunol..

[90] Ruterbusch M., Pruner K.B., Shehata L., Pepper M. (2020). In Vivo CD4(+) T Cell Differentiation and Function: Revisiting the Th1/Th2 Paradigm. Annu. Rev. Immunol..

[91] Wang Z., Liu J., Wang X., Wu Q., Peng Q., Yang T., Sun X., Wang X., Wang Y., Wu W. (2024). Glycosyltransferase B4GALNT1 promotes immunosuppression in hepatocellular carcinoma via the HES4-SPP1-TAM/Th2 axis. Mol. Biomed..

[92] Kogame M., Nagai H., Shinohara M., Igarashi Y., Sumino Y., Ishii K. (2016). Th2 Dominance Might Induce Carcinogenesis in Patients with HCV-Related Liver Cirrhosis. Anticancer. Res..

[93] Shen Y., Xu S., Ye C., Li Q., Chen R., Wu W., Jiang Q., Jia Y., Zhang X., Fan L. (2023). Proteomic and single-cell landscape reveals novel pathogenic mechanisms of HBV-infected intrahepatic cholangiocarcinoma. iScience.

[94] Pan C., Wu Q., Wang S., Mei Z., Zhang L., Gao X., Qian J., Xu Z., Zhang K., Su R. (2022). Combination with Toll-like receptor 4 (TLR4) agonist reverses GITR agonism mediated M2 polarization of macrophage in Hepatocellular carcinoma. Oncoimmunology.

[95] Bian J., Lin J., Long J., Yang X., Yang X., Lu X., Sang X., Zhao H. (2020). T lymphocytes in hepatocellular carcinoma immune microenvironment: Insights into human immunology and immunotherapy. Am. J. Cancer Res..

[96] Wong B.H.S., Poh Z.S., Wei J.T.C., Amuthavalli K., Ho Y.S., Chen S., Mak S.Y., Bi X., Webster R.D., Shelat V.G. (2025). High Extracellular K(+) Skews T-Cell Differentiation Towards Tumour Promoting Th2 and T(reg) Subsets. Eur. J. Immunol..

[97] De Battista D., Zamboni F., Gerstein H., Sato S., Markowitz T.E., Lack J., Engle R.E., Farci P. (2021). Molecular Signature and Immune Landscape of HCV-Associated Hepatocellular Carcinoma (HCC): Differences and Similarities with HBV-HCC. J. Hepatocell. Carcinoma.

[98] Wan J., Wu Y., Ji X., Huang L., Cai W., Su Z., Wang S., Xu H. (2020). IL-9 and IL-9-producing cells in tumor immunity. Cell Commun. Signal.

[99] Rivera Vargas T., Humblin E., Végran F., Ghiringhelli F., Apetoh L. (2017). T(H)9 cells in anti-tumor immunity. Semin. Immunopathol..

[100] Chen J., Guan L., Tang L., Liu S., Zhou Y., Chen C., He Z., Xu L. (2019). T Helper 9 Cells: A New Player in Immune-Related Diseases. DNA Cell Biol..

[101] Elyaman W., Bradshaw E.M., Uyttenhove C., Dardalhon V., Awasthi A., Imitola J., Bettelli E., Oukka M., van Snick J., Renauld J.C. (2009). IL-9 induces differentiation of TH17 cells and enhances function of FoxP3+ natural regulatory T cells. Proc. Natl. Acad. Sci. USA.

[102] Hashemi M., Nadafzadeh N., Imani M.H., Rajabi R., Ziaolhagh S., Bayanzadeh S.D., Norouzi R., Rafiei R., Koohpar Z.K., Raei B. (2023). Targeting and regulation of autophagy in hepatocellular carcinoma: Revisiting the molecular interactions and mechanisms for new therapy approaches. Cell Commun. Signal..

[103] Tan H., Wang S., Zhao L. (2017). A tumour-promoting role of Th9 cells in hepatocellular carcinoma through CCL20 and STAT3 pathways. Clin. Exp. Pharmacol. Physiol..

[104] Hou K.Z., Fu Z.Q., Gong H. (2015). Chemokine ligand 20 enhances progression of hepatocellular carcinoma via epithelial-mesenchymal transition. World J. Gastroenterol..

[105] Li H.J., Sun Q.M., Liu L.Z., Zhang J., Huang J., Wang C.H., Ding R., Song K., Tong Z. (2015). High expression of IL-9R promotes the progression of human hepatocellular carcinoma and indicates a poor clinical outcome. Oncol. Rep..

[106] Hou C.Y., Lv P., Yuan H.F., Zhao L.N., Wang Y.F., Zhang H.H., Yang G., Zhang X.D. (2024). Bevacizumab induces ferroptosis and enhances CD8(+) T cell immune activity in liver cancer via modulating HAT1 and increasing IL-9. Acta Pharmacol. Sin..

[107] Chen J., Ding Y., Huang F., Lan R., Wang Z., Huang W., Chen R., Wu B., Fu L., Yang Y. (2021). Irradiated whole-cell vaccine suppresses hepatocellular carcinoma growth in mice via Th9 cells. Oncol. Lett..

[108] Miossec P., Kolls J.K. (2012). Targeting IL-17 and TH17 cells in chronic inflammation. Nat. Rev. Drug Discov..

[109] Edwards M., Brockmann L. (2025). Microbiota-dependent modulation of intestinal anti-inflammatory CD4(+) T cell responses. Semin. Immunopathol..

[110] Cho H.J., Cheong J.Y. (2021). Role of Immune Cells in Patients with Hepatitis B Virus-Related Hepatocellular Carcinoma. Int. J. Mol. Sci..

[111] Liu Z., Naz W., Yousaf T., Sun J., Wu Q., Guo M., Tian G. (2025). Viral-Track integrated single-cell RNA-sequencing reveals HBV lymphotropism and immunosuppressive microenvironment in HBV-associated hepatocellular carcinoma. Commun. Biol..

[112] Gomes A.L., Teijeiro A., Burén S., Tummala K.S., Yilmaz M., Waisman A., Theurillat J.P., Perna C., Djouder N. (2016). Metabolic Inflammation-Associated IL-17A Causes Non-alcoholic Steatohepatitis and Hepatocellular Carcinoma. Cancer Cell.

[113] Ma H.Y., Yamamoto G., Xu J., Liu X., Karin D., Kim J.Y., Alexandrov L.B., Koyama Y., Nishio T., Benner C. (2020). IL-17 signaling in steatotic hepatocytes and macrophages promotes hepatocellular carcinoma in alcohol-related liver disease. J. Hepatol..

[114] Gasmi I., Machou C., Rodrigues A., Brouillet A., Nguyen T.C., Rousseau B., Guillot A., Rodriguez C., Demontant V., Ait-Ahmed Y. (2022). Interleukin-17 programs liver progenitor cell transformation into cancer stem cells through miR-122 downregulation with increased risk of primary liver cancer initiation. Int. J. Biol. Sci..

[115] Sun D., Li W., Ding D., Tan K., Ding W., Wang Z., Fu S., Hou G., Zhou W.P., Gu F. (2024). IL-17a promotes hepatocellular carcinoma by increasing FAP expression in hepatic stellate cells via activation of the STAT3 signaling pathway. Cell Death Discov..

[116] Gu F.M., Li Q.L., Gao Q., Jiang J.H., Zhu K., Huang X.Y., Pan J.F., Yan J., Hu J.H., Wang Z. (2011). IL-17 induces AKT-dependent IL-6/JAK2/STAT3 activation and tumor progression in hepatocellular carcinoma. Mol. Cancer.

[117] Xu Q.G., Yu J., Guo X.G., Hou G.J., Yuan S.X., Yang Y., Yang Y., Liu H., Pan Z.Y., Yang F. (2018). IL-17A promotes the invasion-metastasis cascade via the AKT pathway in hepatocellular carcinoma. Mol. Oncol..

[118] Jiang R., Tan Z., Deng L., Chen Y., Xia Y., Gao Y., Wang X., Sun B. (2011). Interleukin-22 promotes human hepatocellular carcinoma by activation of STAT3. Hepatology.

[119] Jia W., Liang S., Lin W., Li S., Yuan J., Jin M., Nie S., Zhang Y., Zhai X., Zhou L. (2023). Hypoxia-induced exosomes facilitate lung pre-metastatic niche formation in hepatocellular carcinoma through the miR-4508-RFX1-IL17A-p38 MAPK-NF-κB pathway. Int. J. Biol. Sci..

[120] Kuang D.M., Peng C., Zhao Q., Wu Y., Chen M.S., Zheng L. (2010). Activated monocytes in peritumoral stroma of hepatocellular carcinoma promote expansion of memory T helper 17 cells. Hepatology.

[121] Zhao Q., Xiao X., Wu Y., Wei Y., Zhu L.Y., Zhou J., Kuang D.M. (2011). Interleukin-17-educated monocytes suppress cytotoxic T-cell function through B7-H1 in hepatocellular carcinoma patients. Eur. J. Immunol..

[122] Song M., Wang L., Jiang S., Liang J., Li W., Rao W., Du Q., Liu G., Meng H., Tang L. (2024). Pathogenic Th17 cell-mediated liver fibrosis contributes to resistance to PD-L1 antibody immunotherapy in hepatocellular carcinoma. Int. Immunopharmacol..

[123] Zhao F., Hoechst B., Gamrekelashvili J., Ormandy L.A., Voigtländer T., Wedemeyer H., Ylaya K., Wang X.W., Hewitt S.M., Manns M.P. (2012). Human CCR4+ CCR6+ Th17 cells suppress autologous CD8+ T cell responses. J. Immunol..

[124] Wang W., Wang Z., Qin Y., Tang G., Cai G., Liu Y., Zhang J., Zhang P., Shen Q., Shen L. (2018). Th17, synchronically increased with T(regs) and B(regs), promoted by tumour cells via cell-contact in primary hepatic carcinoma. Clin. Exp. Immunol..

[125] Liao R., Sun J., Wu H., Yi Y., Wang J.X., He H.W., Cai X.Y., Zhou J., Cheng Y.F., Fan J. (2013). High expression of IL-17 and IL-17RE associate with poor prognosis of hepatocellular carcinoma. J. Exp. Clin. Cancer Res..

[126] Ren H., Chen Y., Zhu Z., Xia J., Liu S., Hu Y., Qin X., Zhang L., Ding Y., Xia S. (2023). FOXO1 regulates Th17 cell-mediated hepatocellular carcinoma recurrence after hepatic ischemia-reperfusion injury. Cell Death Dis..

[127] Seth P., Dubey S. (2023). IL-22 as a target for therapeutic intervention: Current knowledge on its role in various diseases. Cytokine.

[128] Trifari S., Kaplan C.D., Tran E.H., Crellin N.K., Spits H. (2009). Identification of a human helper T cell population that has abundant production of interleukin 22 and is distinct from T(H)-17, T(H)1 and T(H)2 cells. Nat. Immunol..

[129] Zhang T., Wahib R., Zazara D.E., Lücke J., Shiri A.M., Kempski J., Zhao L., Agalioti T., Machicote A.P., Giannou O. (2023). CD4+ T cell-derived IL-22 enhances liver metastasis by promoting angiogenesis. Oncoimmunology.

[130] Chen J., Sun S., Li H., Cai X., Wan C. (2024). IL-22 signaling promotes sorafenib resistance in hepatocellular carcinoma via STAT3/CD155 signaling axis. Front. Immunol..

[131] Mansur F., Arshad T., Liska V., Manzoor S. (2023). Interleukin-22 promotes the proliferation and migration of hepatocellular carcinoma cells via the phosphoinositide 3-kinase (PI3K/AKT) signaling pathway. Mol. Biol. Rep..

[132] Qin S., Ma S., Huang X., Lu D., Zhou Y., Jiang H. (2014). Th22 cells are associated with hepatocellular carcinoma development and progression. Chin. J. Cancer Res..

[133] Lücke J., Sabihi M., Zhang T., Bauditz L.F., Shiri A.M., Giannou A.D., Huber S. (2021). The good and the bad about separation anxiety: Roles of IL-22 and IL-22BP in liver pathologies. Semin. Immunopathol..

[134] Kuang D.M., Xiao X., Zhao Q., Chen M.M., Li X.F., Liu R.X., Wei Y., Ouyang F.Z., Chen D.P., Wu Y. (2014). B7-H1-expressing antigen-presenting cells mediate polarization of protumorigenic Th22 subsets. J. Clin. Investig..

[135] Gutiérrez-Melo N., Baumjohann D. (2023). T follicular helper cells in cancer. Trends Cancer.

[136] Zeng X., Pan Y., Lin J., Zheng Z., Wu H., Wang Y., Wu Y., Shen Y., Chen Y., Zhao Y. (2025). IL-21R-Targeted Nano-immunosuppressant Prevents Acute Rejection in Allogeneic Transplantation by Blocking Maturation of T Follicular Helper Cells. Acta Biomater..

[137] Zhang H., Zheng H., Wang Y., Chen C., Tong Y., Xie S., Ma X., Guo L., Lu R. (2025). PD-1 suppresses human CD38(+) circulating Tfr cells and regulates humoral immunity. J. Immunother. Cancer.

[138] Zhou Z.Q., Tong D.N., Guan J., Tan H.W., Zhao L.D., Zhu Y., Yao J., Yang J., Zhang Z.Y. (2016). Follicular helper T cell exhaustion induced by PD-L1 expression in hepatocellular carcinoma results in impaired cytokine expression and B cell help, and is associated with advanced tumor stages. Am. J. Transl. Res..

[139] Kurebayashi Y., Sugimoto K., Tsujikawa H., Matsuda K., Nomura R., Ueno A., Masugi Y., Yamazaki K., Effendi K., Takeuchi H. (2024). Spatial Dynamics of T- and B-Cell Responses Predicts Clinical Outcome of Resectable and Unresectable Hepatocellular Carcinoma. Clin. Cancer Res..

[140] Calderaro J., Petitprez F., Becht E., Laurent A., Hirsch T.Z., Rousseau B., Luciani A., Amaddeo G., Derman J., Charpy C. (2019). Intra-tumoral tertiary lymphoid structures are associated with a low risk of early recurrence of hepatocellular carcinoma. J. Hepatol..

[141] Ding G.Y., Ma J.Q., Yun J.P., Chen X., Ling Y., Zhang S., Shi J.Y., Chang Y.Q., Ji Y., Wang X.Y. (2022). Distribution and density of tertiary lymphoid structures predict clinical outcome in intrahepatic cholangiocarcinoma. J. Hepatol..

[142] Wang B., Zhu J., Ma X., Qiu S., Pan B., Zhou J., Fan J., Yang X., Guo W. (2020). Tfr-Tfh index: A new predicator for recurrence of hepatocellular carcinoma patients with HBV infection after curative resection. Clin. Chim. Acta.

[143] Chen M.M., Xiao X., Lao X.M., Wei Y., Liu R.X., Zeng Q.H., Wang J.C., Ouyang F.Z., Chen D.P., Chan K.W. (2016). Polarization of Tissue-Resident TFH-Like Cells in Human Hepatoma Bridges Innate Monocyte Inflammation and M2b Macrophage Polarization. Cancer Discov..

[144] Li M., Wang L., Cong L., Wong C.C., Zhang X., Chen H., Zeng T., Li B., Jia X., Huo J. (2024). Spatial proteomics of immune microenvironment in nonalcoholic steatohepatitis-associated hepatocellular carcinoma. Hepatology.

[145] Zhang L., Han C., Shrestha M.M., Le J., Berger W.K., Huang Y., Desrouleaux R., Wang E., Nagy L., Yang X. (2025). The OGT-TFF2 axis mediates intrahepatic crosstalk and MASH pathogenesis. Hepatology.

[146] Fu J.T., Liu J., Wu W.B., Chen Y.T., Lu G.D., Cao Q., Meng H.B., Tong J., Zhu J.H., Wang X.J. (2024). Targeting EFHD2 inhibits interferon-γ signaling and ameliorates non-alcoholic steatohepatitis. J. Hepatol..

[147] Rodriguez E., Simon P., Dhooge S., Fernandez M., Calafat P., Kurpis M., Nuñez N., Prieto J., Saborowski A., Vogel A. (2026). Hepatic CD4 T Cells Predict Hepatocellular Carcinoma Risk on Metabolic Dysfunction-Associated Steatohepatitis Patients. United Eur. Gastroenterol. J..

[148] Zheng Y., Zhao L., Xiong Z., Huang C., Yong Q., Fang D., Fu Y., Gu S., Chen C., Li J. (2024). Ursolic acid targets secreted phosphoprotein 1 to regulate Th17 cells against metabolic dysfunction-associated steatotic liver disease. Clin. Mol. Hepatol..

[149] Ren Y., Liu X., Feng M., Zhao J., Duan Y., Dong G., Gao H., Hao X., Wang Q., Yao J. (2025). HIVEP1 aggravates NASH by reprogramming polyamine metabolism in T(H)17 cells. Sci. Transl. Med..

[150] Dywicki J., Buitrago-Molina L.E., Baumann A.K., Davalos-Misslitz A.C., Hendriks C.M., Hupa-Breier K.L., Lieber M., Schlue J., Blüher M., Bantel H. (2025). From model to man: Understanding Tregs’ dual role in MASLD. JHEP Rep..

[151] Moreno-Fernandez M.E., Giles D.A., Oates J.R., Chan C.C., Damen M., Doll J.R., Stankiewicz T.E., Chen X., Chetal K., Karns R. (2021). PKM2-dependent metabolic skewing of hepatic Th17 cells regulates pathogenesis of non-alcoholic fatty liver disease. Cell Metab..

[152] Zhang H., Ma Y., Cheng X., Wu D., Huang X., Chen B., Ren Y., Jiang W., Tang X., Bai T. (2021). Targeting epigenetically maladapted vascular niche alleviates liver fibrosis in nonalcoholic steatohepatitis. Sci. Transl. Med..

[153] Ma C., Kesarwala A.H., Eggert T., Medina-Echeverz J., Kleiner D.E., Jin P., Stroncek D.F., Terabe M., Kapoor V., ElGindi M. (2016). NAFLD causes selective CD4(+) T lymphocyte loss and promotes hepatocarcinogenesis. Nature.

[154] Savage T.M., Fortson K.T., de Los Santos-Alexis K., Oliveras-Alsina A., Rouanne M., Rae S.S., Gamarra J.R., Shayya H., Kornberg A., Cavero R. (2024). Amphiregulin from regulatory T cells promotes liver fibrosis and insulin resistance in non-alcoholic steatohepatitis. Immunity.

[155] Huang P., Rodriguez-Matos F.J., Qi J., Trehan R., Myojin Y., Zhu X.B., Greten T.F., Ma C. (2025). Hepatic immune environment differences among common mouse strains in models of MASH and liver cancer. JHEP Rep..

[156] Huang Y., Xie Y., Zhang Y., Liu Z., Jiang W., Ye Y., Tang J., Li Z., Yin Z., Lin X.J. (2025). Single-cell transcriptome reveals the reprogramming of immune microenvironment during the transition from MASH to HCC. Mol. Cancer.

[157] Rolla S., Alchera E., Imarisio C., Bardina V., Valente G., Cappello P., Mombello C., Follenzi A., Novelli F., Carini R. (2016). The balance between IL-17 and IL-22 produced by liver-infiltrating T-helper cells critically controls NASH development in mice. Clin. Sci..

[158] Jin J., Cheng K., Chen M., Liang H., Zhang W. (2025). Immunotherapy resistance in MASLD-related hepatocellular carcinoma: Special immune microenvironment and gut microbiota. Int. J. Biol. Sci..

[159] Budhu A., Forgues M., Ye Q.H., Jia H.L., He P., Zanetti K.A., Kammula U.S., Chen Y., Qin L.X., Tang Z.Y. (2006). Prediction of venous metastases, recurrence, and prognosis in hepatocellular carcinoma based on a unique immune response signature of the liver microenvironment. Cancer Cell.

[160] Wu K., Zhang G., Shen C., Zhu L., Yu C., Sartorius K., Ding W., Jiang Y., Lu Y. (2024). Role of T cells in liver metastasis. Cell Death Dis..

[161] Gu J., Yu Z., Tang X., Chen W., Deng X., Zhu X. (2024). Cryoablation combined with dual immune checkpoint blockade enhances antitumor efficacy in hepatocellular carcinoma model mice. Int. J. Hyperth..

[162] He X., Li X., Liu B., Xu L., Zhao H., Lu A. (2011). Down-regulation of Treg cells and up-regulation of TH1/TH2 cytokine ratio were induced by polysaccharide from Radix Glycyrrhizae in H22 hepatocarcinoma bearing mice. Molecules.

[163] Jiayi C., Siru C., Xiaoqi L., Enling X., Hui W., Juze L., Changjun W. (2024). Effects of Jianpi Huayu Decoction on Th1/Th2 Immune Balance in Mice with Liver Cancer-Related Fatigue via the IL- 27/STAT1 Signaling Pathway. Integr. Cancer Ther..

[164] Lin J., Huang J., Tan C., Wu S., Lu X., Pu J. (2024). LncRNA MEG3 suppresses hepatocellular carcinoma by stimulating macrophage M1 polarization and modulating immune system via inhibiting CSF-1 in vivo/vitro studies. Int. J. Biol. Macromol..

[165] Zhu Y., Yang J., Xu D., Gao X.M., Zhang Z., Hsu J.L., Li C.W., Lim S.O., Sheng Y.Y., Zhang Y. (2019). Disruption of tumour-associated macrophage trafficking by the osteopontin-induced colony-stimulating factor-1 signalling sensitises hepatocellular carcinoma to anti-PD-L1 blockade. Gut.

[166] Cardenas M.A., Prokhnevska N., Sobierajska E., Gregorova P., Medina C.B., Valanparambil R.M., Greenwald R., DelBalzo L., Bilen M.A., Joshi S.S. (2024). Differentiation fate of a stem-like CD4 T cell controls immunity to cancer. Nature.

[167] Huang Y., Wang F., Wang Y., Zhu Z., Gao Y., Ma Z., Xu R., Du Z. (2014). Intrahepatic interleukin-17+ T cells and FoxP3+ regulatory T cells cooperate to promote development and affect the prognosis of hepatocellular carcinoma. J. Gastroenterol. Hepatol..

[168] Mou H., Wu S., Zhao G., Wang J. (2019). Changes of Th17/Treg ratio in the transition of chronic hepatitis B to liver cirrhosis and correlations with liver function and inflammation. Exp. Ther. Med..

[169] Xu L., Kitani A., Fuss I., Strober W. (2007). Cutting edge: Regulatory T cells induce CD4+CD25-Foxp3- T cells or are self-induced to become Th17 cells in the absence of exogenous TGF-beta. J. Immunol..

[170] Kleinewietfeld M., Hafler D.A. (2013). The plasticity of human Treg and Th17 cells and its role in autoimmunity. Semin. Immunol..

[171] Zhou L., Lopes J.E., Chong M.M., Ivanov I.I., Min R., Victora G.D., Shen Y., Du J., Rubtsov Y.P., Rudensky A.Y. (2008). TGF-beta-induced Foxp3 inhibits T(H)17 cell differentiation by antagonizing RORgammat function. Nature.

[172] Downs-Canner S., Berkey S., Delgoffe G.M., Edwards R.P., Curiel T., Odunsi K., Bartlett D.L., Obermajer N. (2017). Suppressive IL-17A(+)Foxp3(+) and ex-Th17 IL-17A(neg)Foxp3(+) T(reg) cells are a source of tumour-associated T(reg) cells. Nat. Commun..

[173] Groneberg M., Hoenow S., Marggraff C., Fehling H., Metwally N.G., Hansen C., Bruchhaus I., Tiegs G., Sellau J., Lotter H. (2022). HIF-1α modulates sex-specific Th17/Treg responses during hepatic amoebiasis. J. Hepatol..

[174] Leone V., Ali A., Weber A., Tschaharganeh D.F., Heikenwalder M. (2021). Liver Inflammation and Hepatobiliary Cancers. Trends Cancer.

[175] Liu B., Gao W., Zhang L., Wang J., Chen M., Peng M., Ren H., Hu P. (2017). Th17/Treg imbalance and increased interleukin-21 are associated with liver injury in patients with chronic severe hepatitis B. Int. Immunopharmacol..

[176] Li K., Liu H., Guo T. (2017). Th17/Treg imbalance is an indicator of liver cirrhosis process and a risk factor for HCC occurrence in HBV patients. Clin. Res. Hepatol. Gastroenterol..

[177] Yu S.J., Jiang R., Mazzu Y.Z., Wei C.B., Sun Z.L., Zhang Y.Z., Zhou L.D., Zhang Q.H. (2016). Epigallocatechin-3-gallate Prevents Triptolide-Induced Hepatic Injury by Restoring the Th17/T(reg) Balance in Mice. Am. J. Chin. Med..

[178] Zhang H., Yuan Z., Zhu Y., Yuan Z., Wang J., Nong C., Zhou S., Tang Q., Zhang L., Jiang Z. (2022). Th17/Treg imbalance mediates hepatic intolerance to exogenous lipopolysaccharide and exacerbates liver injury in triptolide induced excessive immune response. J. Ethnopharmacol..

[179] Yao C., Lan D., Li X., Wang Y., Qi S., Liu Y. (2023). Porphyromonas gingivalis is a risk factor for the development of nonalcoholic fatty liver disease via ferroptosis. Microbes Infect..

[180] Li L., Xia Y., Ji X., Wang H., Zhang Z., Lu P., Ding Q., Wang D., Liu M. (2021). MIG/CXCL9 exacerbates the progression of metabolic-associated fatty liver disease by disrupting Treg/Th17 balance. Exp. Cell Res..

[181] Liu J., Li W., Zhu W., He W., Zhao H., Xiang Y., Liu C., Wu W. (2018). Chronic intermittent hypoxia promotes the development of experimental non-alcoholic steatohepatitis by modulating Treg/Th17 differentiation. Acta Biochim. Et Biophys. Sin..

[182] Li Y., Jiang H.T., Han L.B., Xiao L., Gan J.H. (2020). MiR-195 regulates CD40 to maintain Th17/Treg balance in rats with non-alcoholic fatty liver disease. Biomed. Pharmacother..

[183] Lan Y.T., Wang Z.L., Tian P., Gong X.N., Fan Y.C., Wang K. (2019). Treg/Th17 imbalance and its clinical significance in patients with hepatitis B-associated liver cirrhosis. Diagn. Pathol..

[184] Lin Z.W., Wu L.X., Xie Y., Ou X., Tian P.K., Liu X.P., Min J., Wang J., Chen R.F., Chen Y.J. (2015). The expression levels of transcription factors T-bet, GATA-3, RORγt and FOXP3 in peripheral blood lymphocyte (PBL) of patients with liver cancer and their significance. Int. J. Med. Sci..

[185] Nwabo Kamdje A.H., Tagne Simo R., Fogang Dongmo H.P., Bidias A.R., Masumbe Netongo P. (2023). Role of signaling pathways in the interaction between microbial, inflammation and cancer. Holist. Integr. Oncol..

[186] Wang J., Hou Y., Mu L., Yang M., Ai X. (2024). Gut microbiota contributes to the intestinal and extraintestinal immune homeostasis by balancing Th17/Treg cells. Int. Immunopharmacol..

[187] Song J., Dai J., Chen X., Ding F., Ding Y., Ma L., Zhang L. (2024). Bifidobacterium mitigates autoimmune hepatitis by regulating IL-33-induced Treg/Th17 imbalance via the TLR2/4 signaling pathway. Histol. Histopathol..

[188] Chen R.C., Xu L.M., Du S.J., Huang S.S., Wu H., Dong J.J., Huang J.R., Wang X.D., Feng W.K., Chen Y.P. (2016). Lactobacillus rhamnosus GG supernatant promotes intestinal barrier function, balances Treg and TH17 cells and ameliorates hepatic injury in a mouse model of chronic-binge alcohol feeding. Toxicol. Lett..

[189] Saeedi B.J., Liu K.H., Owens J.A., Hunter-Chang S., Camacho M.C., Eboka R.U., Chandrasekharan B., Baker N.F., Darby T.M., Robinson B.S. (2020). Gut-Resident Lactobacilli Activate Hepatic Nrf2 and Protect Against Oxidative Liver Injury. Cell Metab..

[190] Zhou D., Pan Q., Liu X.L., Yang R.X., Chen Y.W., Liu C., Fan J.G. (2017). Clostridium butyricum B1 alleviates high-fat diet-induced steatohepatitis in mice via enterohepatic immunoregulation. J. Gastroenterol. Hepatol..

[191] Huo R., Yang W.J., Liu Y., Liu T., Li T., Wang C.Y., Pan B.S., Wang B.L., Guo W. (2024). Stigmasterol: Remodeling gut microbiota and suppressing tumor growth through Treg and CD8+ T cells in hepatocellular carcinoma. Phytomedicine.

[192] Cui D., Zhang C., Zhang L., Zheng J., Wang J., He L., Jin H., Kang Q., Zhang Y., Li N. (2025). Natural anti-cancer products: Insights from herbal medicine. Chin. Med..

[193] Chen T.T., Du S.L., Wang S.J., Wu L., Yin L. (2022). Dahuang Zhechong pills inhibit liver cancer growth in a mouse model by reversing Treg/Th1 balance. Chin. J. Nat. Med..

[194] Wu L., Yang F.R., Xing M.L., Lu S.F., Chen H.L., Yang Q.W., Zhang Y.T., Lu Y., Huang Y. (2022). Multi-material basis and multi-mechanisms of the Dahuang Zhechong pill for regulating Treg/Th1 balance in hepatocellular carcinoma. Phytomedicine.

[195] Xie Y., Zhang Y., Wei X., Zhou C., Huang Y., Zhu X., Chen Y., Wen H., Huang X., Lin J. (2020). Jianpi Huayu Decoction Attenuates the Immunosuppressive Status of H(22) Hepatocellular Carcinoma-Bearing Mice: By Targeting Myeloid-Derived Suppressor Cells. Front. Pharmacol..

[196] Kan X., Zhang W., You R., Niu Y., Guo J., Xue J. (2017). Scutellaria barbata D. Don extract inhibits the tumor growth through down-regulating of Treg cells and manipulating Th1/Th17 immune response in hepatoma H22-bearing mice. BMC Complement. Altern. Med..

[197] Pan B., Yao Y., Wu H., Ye D., Zhang Z., Zhang X., Wang X., Tang N. (2025). N-glycosylated LTβR increases the Th17/Treg cell ratio in liver cancer by blocking RORC ubiquitination and FOXP3 transcription. Cell Death Dis..

[198] Chen Y., Dai S., Cheng C.S., Chen L. (2024). Lenvatinib and immune-checkpoint inhibitors in hepatocellular carcinoma: Mechanistic insights, clinical efficacy, and future perspectives. J. Hematol. Oncol..

[199] Tian B., Wang Z., Cao M., Wang N., Jia X., Zhang Y., Zhou J., Liu S., Zhang W., Dong X. (2025). CCR8 antagonist suppresses liver cancer progression via turning tumor-infiltrating Tregs into less immunosuppressive phenotype. J. Exp. Clin. Cancer Res..

[200] Sun R., Lee K.Y., Mei Y., Nickles E., Le Lin J., Xia R., Liu H., Schwarz H. (2025). Induction of cell death in malignant cells and regulatory T cells in the tumor microenvironment by targeting CD137. Oncoimmunology.

[201] Lin Z., Jiang C., Wang P., Chen Q., Wang B., Fu X., Liang Y., Zhang D., Zeng Y., Liu X. (2023). Caveolin-mediated cytosolic delivery of spike nanoparticle enhances antitumor immunity of neoantigen vaccine for hepatocellular carcinoma. Theranostics.

[202] Liu N., Chang C.W., Steer C.J., Wang X.W., Song G. (2022). MicroRNA-15a/16-1 Prevents Hepatocellular Carcinoma by Disrupting the Communication Between Kupffer Cells and Regulatory T Cells. Gastroenterology.

[203] Liu N., Steer C.J., Song G. (2022). MicroRNA-206 enhances antitumor immunity by disrupting the communication between malignant hepatocytes and regulatory T cells in c-Myc mice. Hepatology.

[204] Wu X., Xue R., Peng H., Gan X., Lu X., Yan W., Tian Y., Ni X., Shen H., Cheng F. (2019). Traf6 inhibitor boosts antitumor immunity by impeding regulatory T cell migration in Hepa1-6 tumor model. Int. Immunopharmacol..

[205] Zhang Q., Huang H., Zheng F., Liu H., Qiu F., Chen Y., Liang C.L., Dai Z. (2020). Resveratrol exerts antitumor effects by downregulating CD8(+)CD122(+) Tregs in murine hepatocellular carcinoma. Oncoimmunology.

[206] Qiao X., Ma P., Guo J., Sun Y., Peng L., Yu N., Zuo W., Yang J. (2025). Transforming “cold” tumors into “hot” ones via immunogenic cell death by bufadienolides for reverse immunosuppressive TME in HCC. J. Mater. Chem. B.

[207] Gao Y., You M., Fu J., Tian M., Zhong X., Du C., Hong Z., Zhu Z., Liu J., Markowitz G.J. (2022). Intratumoral stem-like CCR4+ regulatory T cells orchestrate the immunosuppressive microenvironment in HCC associated with hepatitis B. J. Hepatol..

[208] Hochnadel I., Hoenicke L., Petriv N., Neubert L., Reinhard E., Hirsch T., Alfonso J.C.L., Suo H., Longerich T., Geffers R. (2022). Safety and efficacy of prophylactic and therapeutic vaccine based on live-attenuated Listeria monocytogenes in hepatobiliary cancers. Oncogene.

[209] Sang L., Li J., Zhang F., Jia J., Zhang J., Ding P., Sun T., Wang D. (2022). Glycyrrhetinic acid modified chlorambucil prodrug for hepatocellular carcinoma treatment based on DNA replication and tumor microenvironment. Colloids Surf. B Biointerfaces.

[210] Guo K., Bu L., Du J., Zhang W., Xia J., Tao M., Shao X., Liu L., Zhao W., Cai Y. (2025). A novel ionizable lipid with comprehensive improvements in transfection potency, immune profile and safety of lipid nanoparticle. J. Control. Release.

[211] Sugai S., Yoshikawa T., Iwama T., Tsuchiya N., Ueda N., Fujinami N., Shimomura M., Zhang R., Kaneko S., Uemura Y. (2016). Hepatocellular carcinoma cell sensitivity to Vγ9Vδ2 T lymphocyte-mediated killing is increased by zoledronate. Int. J. Oncol..

[212] Shao F., Zhang M., Xu L., Yin D., Li M., Jiang Q., Zhang Q., Yang Y. (2020). Multiboosting of Cancer Immunotherapy by a Core-Shell Delivery System. Mol. Pharm..

[213] Zhao D., Long X.D., Lu T.F., Wang T., Zhang W.W., Liu Y.X., Cui X.L., Dai H.J., Xue F., Xia Q. (2015). Metformin decreases IL-22 secretion to suppress tumor growth in an orthotopic mouse model of hepatocellular carcinoma. Int. J. Cancer.

[214] Atzeni F., Carriero A., Boccassini L., D’Angelo S. (2021). Anti-IL-17 Agents in the Treatment of Axial Spondyloarthritis. Immunotargets Ther..

[215] Song M., Liang J., Wang L., Li W., Jiang S., Xu S., Tang L., Du Q., Liu G., Meng H. (2023). IL-17A functions and the therapeutic use of IL-17A and IL-17RA targeted antibodies for cancer treatment. Int. Immunopharmacol..

[216] Tang R., Zheng L., Zheng J., Wu J., Chen P., Chen J., Xu D., Zeng Y., Li Q., Zhang Z. (2023). Secukinumab plays a synergistic role with starvation therapy in promoting autophagic cell death of hepatocellular carcinoma via inhibiting IL-17A-increased BCL2 level. Vitr. Cell. Dev. Biol..

[217] Li J., Sung C.Y., Lee N., Ni Y., Pihlajamäki J., Panagiotou G., El-Nezami H. (2016). Probiotics modulated gut microbiota suppresses hepatocellular carcinoma growth in mice. Proc. Natl. Acad. Sci. USA.

[218] Tang Q., Leung J., Peng Y., Sanchez-Fueyo A., Lozano J.J., Lam A., Lee K., Greenland J.R., Hellerstein M., Fitch M. (2022). Selective decrease of donor-reactive T(regs) after liver transplantation limits T(reg) therapy for promoting allograft tolerance in humans. Sci. Transl. Med..

[219] Vienot A., Jacquin M., Rebucci-Peixoto M., Pureur D., Ghiringhelli F., Assenat E., Hammel P., Rosmorduc O., Stouvenot M., Allaire M. (2023). Evaluation of the interest to combine a CD4 Th1-inducer cancer vaccine derived from telomerase and atezolizumab plus bevacizumab in unresectable hepatocellular carcinoma: A randomized non-comparative phase II study (TERTIO-PRODIGE 82). BMC Cancer.

[220] Thomas J.S., Siu L.L., Ingham M., Azad N.S., Meyer C.F., Olszanski A.J., Whalen G.F., Camacho L.H., Hu J.S., Hanna D.L. (2025). Safety and efficacy of intratumourally administered INT230-6 in adult patients with advanced solid tumours: Results from an open-label phase 1/2 dose escalation study. EBioMedicine.

[221] Rimassa L., Chan S.L., Sangro B., Lau G., Kudo M., Reig M., Breder V., Ryu M.-H., Ostapenko Y., Sukeepaisarnjaroen W. (2025). Five-year overall survival update from the HIMALAYA study of tremelimumab plus durvalumab in unresectable HCC. J. Hepatol..

